# Enhanced anticancer effect of thymidylate synthase dimer disrupters by promoting intracellular accumulation

**DOI:** 10.3389/fphar.2024.1477318

**Published:** 2024-11-11

**Authors:** Gaetano Marverti, Maria Gaetana Moschella, Alice Belardo, Michele Lamesta, Giada Mercanile, Lorenzo Tagliazucchi, Daniele Aiello, Alberto Venturelli, Davide Illuminati, Remo Guerrini, Lorena Losi, Glauco Ponterini, Maria Paola Costi, Domenico D’Arca

**Affiliations:** ^1^ Department of Biomedical, Metabolic and Neural Sciences, University of Modena and Reggio Emilia, Modena, Italy; ^2^ Department of Life Sciences, University of Modena and Reggio Emilia, Modena, Italy; ^3^ Clinical and Experimental Medicine PhD Program (CEM), University of Modena and Reggio Emilia, Modena, Italy; ^4^ Department of Medical and Surgical Sciences for Children and Adults, University of Modena and Reggio Emilia, Modena, Italy; ^5^ Department of Precision Medicine in Medical, Surgical and Critical Care (Me.Pre.C.C.) PhD in Precision Medicine, University of Palermo, Palermo, Italy; ^6^ Department of Chemical and Pharmaceutical Science, University of Ferrara, Ferrara, Italy

**Keywords:** thymidylate synthase, dimer disrupters, SAINT-protein, ABC cassette, cell cycle, synergy quotient

## Abstract

**Introduction:**

Thymidylate synthase (TS) plays a crucial role in cellular growth, proliferation, DNA synthesis, and repair, thus gaining attention for targeted therapies in cancer. TS overexpression and the altered pharmacokinetics of anti-TS drugs are among the most prominent causes of cellular resistance. Decreased drug influx and/or efficient efflux result in reduced drug access to the intracellular targets.

**Results:**

In this study, we have evaluated and demonstrated the increased cytotoxic efficacy of novel TS dimer disrupters (Ddis) in the presence of specific inhibitors of drug efflux protein pumps in ovarian and colon cancer cells, suggesting that these compounds are substrates of the cellular drug extruders. A second strategy adopted to favor intracellular accumulation was to employ, as a drug delivery system, a molecular tool able to help less lipophilic compounds to cross the cell membrane. The Ddis were delivered through the SAINT-Protein transfection agent. The observed cell-killing effects agreed with the reduction of TS protein level and cell cycle perturbation.

**Conclusion:**

Overall, this preclinical study suggests that the innovative TS dimer disrupters can be optimized by increasing their intracellular accumulation by both inhibiting their outflow and/or enhancing cellular uptake.

## 1 Introduction

Thymidylate synthase (TS) catalyzes the reductive methylation of 2′-deoxyuridine-5′-monophosphate (dUMP) by N^5^,N^10^-methylenetetrahydrofolate, (MTHF) CH2H4PteGlu, generating 2′-deoxythymidine-5′-monophosphate (dTMP) and dihydrofolate (Carreras and Santi, 1995). This reaction is essential in the biosynthesis of nucleotide bases of DNA, thus an important target for chemotherapy (Wilson et al., 2014; Taddia et al., 2015). The human TS protein (*h*TS, EC 2.1.1.45) is a highly conserved homodimer of 35 kDa subunits which exerts tight control with a negative autoregulatory mechanism over its synthesis during the cell cycle by binding to TS mRNA, thereby regulating translational processing of the message (Chu and Allegra, 1996). However, several studies with cultured cell lines, tumour models, and clinical specimens, have demonstrated that TS active site inhibitors show restoration of the translational efficiency of the mRNA (Keyomarsi et al., 1993; Yin et al., 2017), induce enzyme levels by about 2–4-fold. This provides the basis for the so-called translational autoregulation model and accounts for the occurrence of resistance to TS-targeted compounds such as 5-fluorodeoxyuridine-5′-monophosphate (FdUMP), which is metabolically derived by the prodrug 5-fluorouracil (5-FU) (Chu and Allegra, 1996). The overexpression of TS is not the only mechanism of drug resistance in TS-targeted treatment, which also includes an altered expression of some other enzymes related to the folate cycle including dihydrofolate reductase (DHFR), glycinamide ribonucleotide formyl-transferase (GARFT), serine hydroxymethyl transferase (SHMT) and dihydropyrimidine dehydrogenase (DPD), making cancer cells resistant to antimetabolites and fluoropyrimidines (Taddia et al., 2015; D’Arca et al., 2023). In addition, the appropriate amount of drug on intracellular target is often limited by reduced drug uptake and/or increased drug extrusion by the resistant cancer cells (Wang et al., 2019). Among the most relevant mechanisms responsible of the multidrug resistance (MDR) development in clinical setting, is the overexpression of extrusion pumps including multidrug resistance gene 1 (MDR1) and corresponding P-glycoprotein protein (P-gp) that severely limits the success of chemotherapy in ovarian cancer treatment (Guppy et al., 2005), statistically shortening the overall survival of ovarian cancer patients (Kamazawa et al., 2002). High expression of P-gp has been also observed at the time of colon cancer diagnosis in patients and is associated with intrinsic resistance to anticancer drugs, as well as it is also inducible by chemotherapeutic agents in colon cell lines, including HCT116 and HT29 (Ding et al., 2010).

The main strategies to overcome drug resistance rely on combination therapies. They are often composed of a TS-targeting compound combined with a drug directed against pathways involved in the resistance phenotype, to achieve additive/synergistic pharmacologic effects. Chemotherapy combined regimens has prolonged the survival of advanced or metastatic cancer patients, by targeting some of the enzymes involved in the metabolic process of 5-fluorouracil (5-FU), such as TS, DPD, and 2′-deoxyuridine-5′triphosphate nucleotidohydrolase (dUTPase), whose inhibition prevents drug catabolism, enhancing the efficacy of TS inhibition by increasing 5-fluorodeoxyuridine-5′-triphosphate (FdUTP) incorporation into DNA (Wilson et al., 2014). Some other strategies rely on the modulation of pathways involved in the repair of DNA damage as the BER (Base excision repair) pathway induced by TS-targeted chemotherapy represents an attractive target for improving drug efficacy (Bulgar et al., 2012).

**FIGURE 1:**
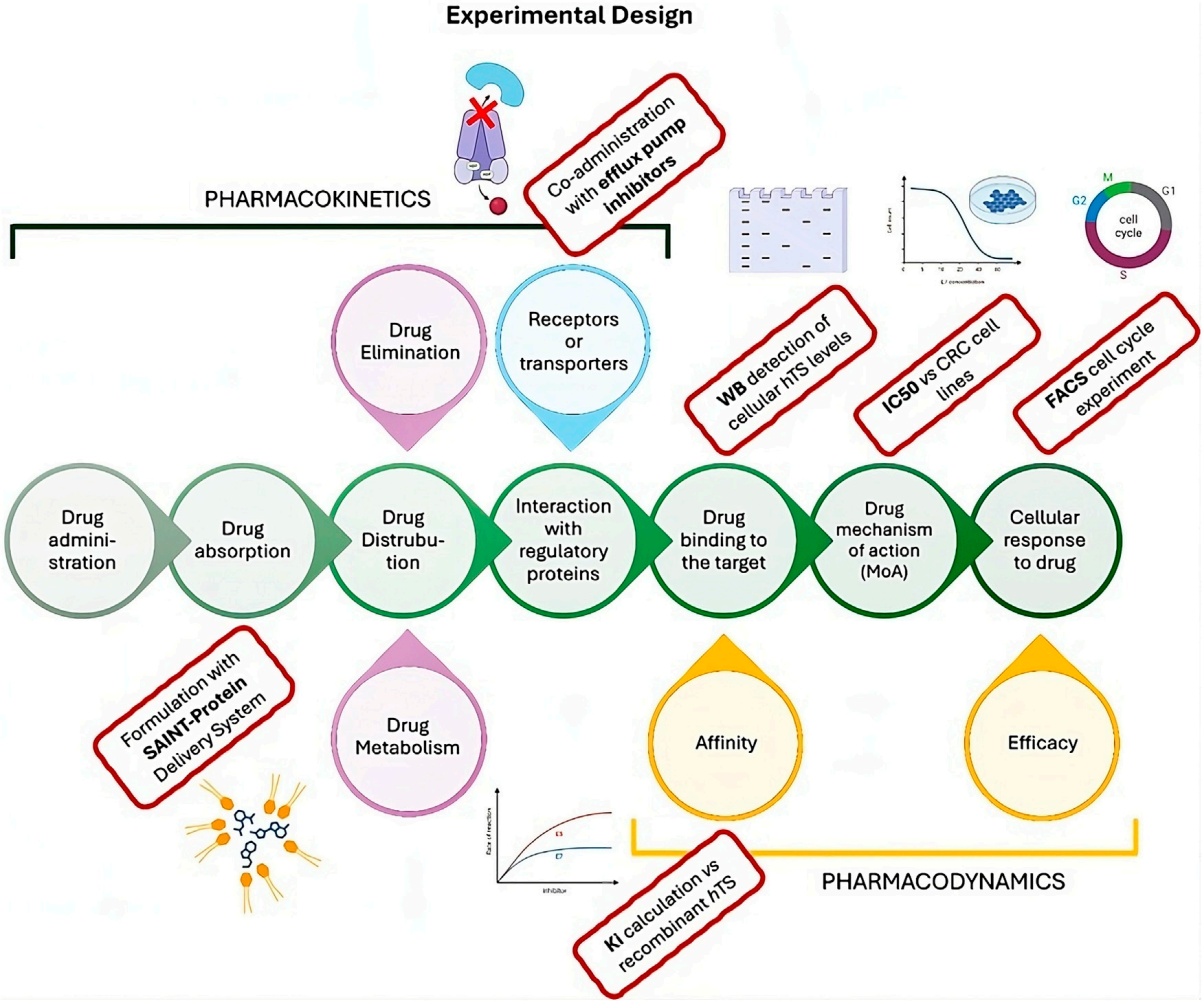
Cellular pharmacokinetic and pharmacodynamic process (central green circles) showing the experimental design of the present work. The experimental steps performed in this paper to characterize the Ddis activity are reported in the red rectangles.

Among the novel strategies to overcome resistance mechanisms towards TS- and TS-pathway-targeted drugs, TS inhibitors binding at allosteric sites and having different mechanism of action relative to active-site binding inhibitors have been actively searched. The monomer-monomer interface of the TS dimer can be regarded as a druggable area for novel inhibitors that, by impairing the dimeric the assembly might successfully inhibit the catalytic function (Cardinale et al., 2011; Pelà et al., 2014; Marverti et al., 2021). We have recently identified several TS-dimer disrupters (Ddis), compounds that bind at the monomer-monomer interface and inhibit the enzyme functions by shifting the dimerization equilibrium of both the recombinant and the intracellular protein toward the inactive monomers (Costantino et al., 2022). These compounds have been shown to engage hTS intracellularly through 4CYS-engineering system and to reduce hTS levels in a dose-dependent manner. Among them, compound **E7** inhibited colorectal and ovarian cancer cell growth and featured an anticancer profile superior to that of **5-FU** in a mouse model of human pancreatic cancer (Costantino et al., 2022). We found that the intracellular mechanism of action of **E7** included TS dimer dissociation to the monomers and faster proteasomal degradation of the latter, resulting in a decrease in the enzyme intracellular level and, thus, in the breaking of the link between TS inhibition and its enhanced expression (Costantino et al., 2022). Other TS Ddis, such as compounds named **E1** (Costantino et al., 2022) (Figure 1) can inhibit the enzyme and destabilize its dimeric assembly but limited cellular-growth inhibition activity, very likely due to inefficient trans-membrane internalization. In this study, we have developed strategies aimed at increasing the intracellular accumulation of the Ddis, by favoring their intracellular retention or by enhancing their internalization process and have characterized the consequent molecular effects by monitoring the *h*TS level changes, cancer cell growth and the apoptotic process.

MDR efflux pumps can efflux compounds from the intracellular to the extracellular compartment. They can bind compounds belonging to different chemical classes and efficiently efflux them out of the cells, thus reducing the intracellular concentration available for the biological effect. These MDR are overexpressed in drug-resistant cancer cells to reduce/inactivate the drug anticancer effect. We have tested specific MDR efflux pump inhibitors in competition with the Ddis compounds to study the capacity of the two MDR inhibitors to enhance cancer cell growth with respect to the administration of the Ddis alone. To this aim, we have considered the third generation P-gp inhibitors, tariquidar and elacridar (GF120918) (Palmeira et al., 2012). Tariquidar displays high-affinity binding to P-gp and shows non-competitive inhibition vs. the P-gp substrates vinblastine and paclitaxel (Li et al., 2015). It abrogates ABCG2 (BCRP)-mediated resistance to camptotecine *in vitro* and potentiates the cytotoxicity of several drugs including doxorubicin, paclitaxel, and etoposide. Co-treatment with vincristine induces a complete reversal of resistance at 25–80 nM (Robey et al., 2004; Shafran et al., 2005). Elacridar is a very potent inhibitor of the ABC transporters (P-glycoprotein, P-gp) and BCRP. It reverses multidrug resistance at 20–100 nM *in vitro*, by blocking the activity of the ABC transporter. Elacridar effectively increases the cellular concentrations and, consequently, the cytotoxicity of anti-tumor drugs such as olaparib and paclitaxel (Vaidyanathan et al., 2016).

As a second strategy favoring the intracellular accumulation of Ddis, consists in transfection of the Ddis using a delivery system. We adopted SAINT-Protein (Synvolux Products and Therapeutics, Leiden, Netherlands), a non-liposomal lipidic reagent used for the intracellular delivery of bioactive substances. SAINT-Protein is a mixture of synthetic lipids with cationic functions forming non-covalent complexes with bioactive compounds. They form positively charged complexes that, by interacting with the phospholipid bilayer of the cellular membrane, are internalized and release the complexed agent inside (Weidner et al., 2016). The internalization process does not involve the endosome that typically is used for the internalization of micelles. This mechanism represents an advantage compared to the endosomal uptake pathway because it represents a rate-limiting barrier for many bioactive compounds (Shete et al., 2014). In membrane trafficking, the endosomal vesicles may trap the bioactive compounds in the lysosomal compartment, making it necessary to adopt effective strategies that facilitate endosomal escape and increase cytosolic bioavailability (Shete et al., 2014). We have recently reported the synergistic antitumor effects of an anti-TS peptide in association with some chemotherapeutic agents delivered into cells both by SAINT-PhD and pH-sensitive pegylated liposomes (Marverti et al., 2020; 2021). Our strategy was to deliver the Ddis compounds inside cells avoiding the possible endo-lysosomal degradation and thus increasing their intracellular concentration and effectiveness. We explore at the cellular level both some pharmacodynamic and pharmacokinetic aspects. The workflow of the performed experiment is represented in Figure 1.

## 2 Materials and methods

### 2.1 Drugs and chemicals

Tariquidar (3-Quinolinecarboxamide, N-[2-[[[4-[2-(3,4-dihydro-6,7-dimethoxy-2(1H)-isoquinolinyl) ethyl]phenyl] amino]carbonyl]-4,5-dimethoxyphenyl]-) and elacridar (N-[4-[2-(6,7-dimethoxy-3,4-dihydro-1H-isoquinolin-2-yl)ethyl]phenyl]-5-methoxy-9-oxo-10H-acridine-4-carbox amide) were purchased from Selleckem (Selleck Chemicals, United States); SAINT-Protein was purchased from Synvolux Products and Therapeutics™ (Leiden, Netherlands). The Ddis compounds **E1** (2-(4-Nitrophenylthio)-N-(4-carboxyphenyl)propionamide), E3 (2-Phenyl-2-(4-nitrophenylthio)-N-(4-carboxyphenyl)carboxyilic acid), **E5** (2-Phenyl-2-(4-nitrophenylthio)-N-(4-carboxamido phenyl)acetamide), **E6** (2-(4-Nitrophenylthio)-N-(3-carbomethoxypyridin-6-yl) acetamide), **E7** (phenyl-2-(4-nitrophenylthio)-N-(6-methanesulfonylbenzothiazol-2-yl)acetamide) were synthesized in the Drug discovery and Biotechnology Lab (P.I. Maria P. Costi, Unimore). Compounds **E1**, **E3** and **E7** were synthesized using modified synthetic procedures reported in SI. Compounds **E5** and **E6** were synthesized as reported (Costantino et al., 2022). The description is reported in the Supplementary Material. The synthesized compounds were identical with respect to those reported in reference (Costantino et al., 2022). **E5**-**E7** were used as racemic mixtures. Compound E5-FITC was synthesized and characterized by Remo Guerrini and Davide Illuminati University of Ferrara (Italy).

### 2.2 Recombinant protein production and characterization

Recombinant 6xHis-thymidylate synthase was expressed in *E. coli* strain BL21 (DE3), purified with affinity and size exclusion FPLC (Akta Prime, GE Healthcare) and characterized with SDS page, MS, and kinetics assays. Briefly, competent *E. coli* previously transformed with pQE80L plasmid encoding for *h*TS were cultured at 37°C in LB Broth, supplemented with 0.1 mM isopropyl β-D-thiogalactopyranoside (IPTG) for 4 h to promote protein expression. Cells were harvested by centrifugation (4,000 g, 30 min, 4°C), resuspended in buffer A (20 mM NaH2PO4 and 30 mM NaCl, pH 7.5) 0.2 mM complete, EDTA-free Protease Inhibitor Cocktail (Roche) and 1 mg mL^−1^ lysozyme, and then disrupted by sonication (four 7s cycles at 70% power, spaced by 10-s pauses, Bandelin Sonoplus HD 3100), after 30 min incubation on ice. After centrifugation (12,000 g, 30 min, 4°C), the soluble fraction was injected into a HisTrap HP 5 mL column (Cytiva) on a FPLC system (Akta Prime, GE Healthcare, Milan, Italy) previously equilibrated with Buffer A. After sample loading a washing step with Buffer B (20 mM NaH_2_PO_4_, 30 mM NaCl, 20 mM imidazole pH 7,5) has been performed, *h*TS was eluted by Buffer C (20 mM NaH_2_PO_4_, 30 mM NaCl, 1 M imidazole, pH 7,5) (Pozzi et al., 2021).

The elution fraction was pooled and furtherly purified from imidazole with 5 mL HiTrap desalting column. The high purity (98%) of the resulting protein sample was confirmed by SDS-PAGE analysis (NuPAGE 4%–12% Bis-Tris protein gels; Thermo Fisher Scientific, Waltham, MA, United States) and High-resolution Mass Spectrometry (Centro Interdipartimentale Grandi Strumenti, University of Modena and Reggio Emilia). (S.I). The enzyme kinetic profile was determined using a spectrophotometric assay. This assay consists of monitoring the absorbance changes at 340 nm for a total time of 180 s. The enzyme activity was measured at a steady-state concentration of N^5^-N^10^-with 75 μM methylenetetrahydrofolate (mTHF) and initiated with 134 μM deoxyuridinemonophosphate (dUMP) (Marverti et al., 2021). The enzyme concentration was varied in a range of 0.5–0.30 μM. The resulting k_cat_ value was determined to be 0.98 ± 0.03 s^−1^. The K_M_ values for mTHF and dUMP substrates were evaluated by varying the corresponding substrate K_M_ values for dUMP and mTHF are 5 and 10 µM respectively. MS characterization of *h*TS is reported in the Supplementary Scheme S3; Supplementary Table S3; Supplementary Figure S3.

### 2.3 Enzyme inhibition

Inhibition assays were performed in a 96-multiwell plate on a Multikskan GO Spectrophotometer (Thermo Fisher Scientific) with increasing inhibitor concentrations in a range depending on compounds solubility (Costantino et al., 2022). Two stock solutions for each inhibitor were prepared by dissolving the compound up to a concentration of 1 mM and 10 mM in DMSO. The different concentration ranges were obtained by serial dilution and added to the reaction mixture, with a % DMSO not exceeding 2% of the total buffer volume. FdUMP was used as reference control and tested at 0-0.1-0.2-0.5-1.0-1.5 µM. 100 nM of 6xHis-tagged *h*TS were incubated in 150 µL of TES buffer (TES buffer 50% v/v (TES/water) (where TES = 2-[[1,3-dihydroxy-2-(hydroxymethyl)propan-2-yl]amino]ethane sulfonic acid), 100 mM; MgCl2 50 mM; Ethylenediaminetetraacetic acid (EDTA) 2 mM; β-mercapto ethanol-(BME) 150 mM; pH 7.4) with the inhibitor at the due concentration. The protein was incubated for 60 min at 37°C under gentle stirring (80rpm). Then mTHF was added to the reaction mixture at a final concentration of 50 µM. The reaction was initiated by the addition of dUMP at a final concentration of 110 µM. A 180 s reading was performed at λ = 340 nm, which corresponds to the maximum absorption of dihydrofolic acid (DHF) resulting from the oxidative demethylation reaction of mTHF.

To make the inhibitory efficacies of the investigated Ddis towards hTS readily comparable with each other, we report the IC_50_ at dUMP and mTHF same concentrations. According to the classical Michaelis-Menten fast-equilibrium enzyme-kinetic equations, the relationship between IC50 and Ki and Ki’, i.e., the equilibrium constants for the dissociations of, respectively, the binary, EI, and ternary, ESI, complexes (E = enzyme, S = substrate, I = inhibitor) depends on the type of inhibition. It is indeed easy to see that, for the general mixed-type inhibition model, IC_50_ = (1+K_M_/[S])/(1/Ki’+K_M_/Ki [S]), K_M_ being the Michaelis constant for the substrate measuring its affinity for the enzyme binding site. For competitive inhibition (Ki’ » IC_50_), IC_50_ = Ki(1+[S]/K_M_) and the IC_50_ is seen to increase with increasing [S]; for non-competitive inhibition, IC_50_ = Ki, i.e., independent of [S]; for the rare case of uncompetitive inhibition (Ki >> IC_50_), IC_50_ = Ki’ (1+K_M_/[S]), asymptotically decreasing to unity with increasing [S]. Thus, for classical inhibition schemes, interpretation of the measured IC_50_ in terms of Ki(Ki’) requires investigation of the inhibition type. This is usually determined together with the Ki/Ki’ in terms of Lineweaver-Burk and double-reciprocal plots. However, the complete dissociative-inhibition model developed in (Costantino et al., 2022) includes three types of Kis, referring to complexes MI, M_2_I and M_2_I_2_, where M is an hTS monomer. The extent to which a Ddis deviates from a competitive inhibitory behaviour, i.e., to which the apparent maximum rate decreases with [I], depends on the abilities of these complexes to act as substrate-subtracting, unproductive or little productive species. We could not solve the dissociative-inhibition model in a closed form amenable to statistical analysis. When forcing simple Lineweaver-Burk and double-reciprocal graphical analysis onto inhibition data obtained with hTS Ddis, we have found different behaviours for different Ddis, interpretable in terms of different abilities of the above complexes to act as unproductive or little productive substrate-subtracting species. Indeed, solution of that model within the fast-equilibrium approximation has provided an IC_50_/Ki ratio (Ki referring to the MI species) that depends on Ki’ (for M_2_I, M_2_I_2_ being there assumed irrelevant), on the enzyme concentration, dimer/monomer equilibrium constant and k_cat_, and on the [S]/K_M_ ratios for dUMP and mTHF (work in progress). So, because the present work focuses on biological rather than chemical-physical features, we have decided, for directly comparing the inhibitory abilities of the tested Ddis, to stick to the phenomenological observable, the IC_50_ value determined from Dixon plots for the same hTS enzyme concentration (100 nM), and in the presence of the same concentrations of dUMP and mTHF ([S]/K_M_ ∼ 10 for both).

### 2.4 Cell cultures

The colon adenocarcinoma HT29 cells and the colon carcinoma HCT116 cells were purchased from ATCC. The human ovarian cancer cell lines A2780, A2780/CP and IGROV-1 are a generous gift of Prof. E. M. Berns, Department of Medical Oncology, Erasmus MC Cancer Institute, Rotterdam, Netherlands, and had been purchased from the European collection of cell cultures, ECACC via Sigma (Beaufort et al., 2014). HT29 and HCT116 (Colorectal Adenocarcinoma cell lines), innately 5FU-resistant cell lines (Bracht et al., 2010) were cultured in Dulbecco Modified Eagle Medium (DMEM), supplemented with 10% heat-inactivated fetal bovine serum and 1% Pen/Strep. A2780 and A2780/CP, ovarian carcinoma cell lines, were cultured in RPMI 1640 medium containing 10% heat-inactivated fetal bovine serum, 1% Pen/Strep. The A2780/CP cells are about 10-fold resistant to cisplatin and derived from the parent A2780 cell line. The IGROV-1 cell line exhibits an epithelial character, highly tumorigenic properties, and a low doubling time (Beaufort et al., 2014). The A2780, IGROV-1 and HT29 are drug-naïve (intrinsic MDR) cell lines. However, HT29 cells express constitutively quite high levels of MDR2 and MDR3 (Yildirim and Aydemir, 2020). Cell lines were incubated at 37°C under 5% CO2 for 24 h before treatments.

The cells under examination were treated 24-h after seeding at a confluence of approximately 50%. The passage number of cell lines used in cell growth and cell cycle analysis were 130 + 5 and unknown + 6 + 5 passages for HT29 and HCT116 cells, respectively. For Western blot analysis, HT29 cells underwent 2 more passages.

For the human ovarian cancer cell lines, the passage number of cell lines used in cell growth and cell cycle analysis were 45 and 50 for A2780 and A2780/CP, respectively, while the IGROV-1 cells underwent 55 passages.

### 2.5 Cell growth evaluation

Cell growth was determined using a modified crystal violet assay (Feoktistova et al., 2016). For the experiments with drug efflux transporter inhibitors, cell lines were pre-treated with 200 nM elacridar or 100 nM tariquidar for 2 h before adding Ddis for 72 h and then processed for cell growth evaluation. For the transfection experiments, cells were treated with drugs transfected or not into cells by means of a previously optimized amount of SAINT-Protein. Transfection of compounds was performed by an adaptation of the standard transfection protocol of the SAINT-Protein delivery system (Synvolux Products, Leiden, NL). Complexes of drugs (2 µL) with SAINT-Protein (8 µL) were prepared in sterile test tubes for the treatment in a 24-well plate without vortexing but gently pipetting 3 times. A slight opalescence indicates optimal mixing of the components. Increasing concentrations of the compounds were added while always maintaining the same volumetric ratio between the drug solutions and the transfectant. Different ratios were less effective and/or resulted in greater nonspecific cytotoxicity. The mixture was incubated for 5 min at room temperature, then filled up to 500 μL with serum-free medium. The culture medium was aspirated from the cells, and the SAINT-Protein/drug complex in serum-free medium was gently added to the wells and incubated for 4 h (37°C; 5% CO2). After this time, a complete medium was added to reach the appropriate volume. All experiments were conducted in duplicate. At the scheduled times, the tissue culture medium was removed, and the cell monolayer was fixed with methanol and stained with 0.2% crystal violet solution in 20% methanol for at least 30 min. After being washed several times with distilled water to remove excess dye, the cells were left to dry. The incorporated dye was solubilized in acidified isopropanol (1N HCl: 2-propanol, 1:10 v/v). After appropriate dilution, the absorbance was measured at 540 nm. The concentration of extracted dye was proportional to the cell number. The percentage of cytotoxicity was calculated by comparing the absorbance of cultures exposed to the drug to that of unexposed (control) cultures (Marverti et al., 2019).

### 2.6 Cell cycle analysis

Cells were treated with drugs transfected or not into cells by means of a previously optimized amount of SAINT-Protein. Transfection of compounds was performed by an adaptation of the standard transfection protocol of the SAINT-Protein delivery system (Synvolux Products, Leiden, NL). Complexes of drugs (5 µL) with SAINT-Protein (20 µL) were prepared in sterile test tubes for the treatment in a 6-well plate without vortexing but gently pipetting 3 times. A slight opalescence indicates optimal mixing of the components. Increasing concentrations of the compounds were added while always maintaining the same volumetric ratio between the drug solutions and the transfectant. Different ratios were less effective and/or resulted in greater nonspecific cytotoxicity. The mixture was incubated for 5 min at room temperature, then filled up to 1,000 μL with serum-free medium. The culture medium was aspirated from the cells, and the SAINT-Protein/drug complex in serum-free medium was gently added to the wells and incubated for 4 h (37°C; 5% CO2). After this time, a complete medium was added to reach the appropriate volume. All experiments were conducted in duplicate. At the scheduled times, the tissue culture medium was removed, and the cell monolayers were harvested. Quantitative measurements of the cell cycle phase distribution were performed by flow cytometry. Cells were then suspended in 0.5 mL of hypotonic fluorochrome solution (50 μg/mL PI, 0.1% sodium citrate, 0.1% Triton X-100). The samples were kept at 4°C in the dark for at least 30 min, dispersed by repeated pipetting before flow cytometry analysis in a FACS Coulter Epics XL flow cytometer equipped with a single 488 nm argon laser. The percentages of nuclei in the different phases of the cell cycle (G0/G1, S and G2/M) were calculated using a DNA cell cycle analysis software (Cell-Fit, Becton Dickinson). A minimum of 10^4^ cells was analyzed for each sample.

### 2.7 Synergy analysis of drugs combination by synergism quotient

The effects of drug combination were quantified by the synergism quotient SQ (Nesterova et al., 2005; Pozzi et al., 2021). SQ was defined as the net growth inhibitory effect of the drug combination divided by the sum of the net individual drug effects on growth inhibition. A quotient> 1 indicates synergism, while a quotient <1 or = 1 indicates an antagonistic or an additive effect, respectively. We considered SAINT-Protein + Ddis compounds as a combination to be evaluated on cancer cells.

### 2.8 Western blot analysis

The cells under examination were seeded in 6-well plates at a confluence of 50%, approximately 500 thousand cells in a final volume of medium corresponding to 3 mL. Once attached, they were treated by adding the compound **E3** (70 µM) directly into the culture medium. **E3** is a compound which, due to its chemical nature, is expected not to cross cell plasma membranes, for this reason, the SAINT-Protein protocol was applied according to the supplier’s directives. The Saint (Protein and Peptide Transfection Reagent, # SP-3004-01 Synvolux Products) should facilitate the internalization of the compounds to be tested against cancer cells. The compounds E3 alone and **E3** + SAINT-Protein were used at the same final concentration of 70 µM. The compounds under investigation were tested for a treatment duration of 48 h. Band intensity was quantified by ImageJ software (Java-based image-processing and analysis software) (Schneider et al., 2012).

Cells were harvested and washed in ice-cold PBS and suspended in RIPA buffer supplemented with protease and phosphatase inhibitors (#78442, Thermo Scientific). The insoluble debris was removed by centrifugation at 14,000 g for 30 min at 4°C. Cellular extracts were resolved using 4%–15% Mini-PROTEAN TGX Gels (#4561083, BIO-RAD) and transferred to Nitrocellulose membranes. Immunoblot analysis was performed using Anti-TYMS Antibody, (clone 1E20 ZooMAb, Sigma Aldrich 1:10,000 dilution), and anti-β-Tubulin (clone AA2, Merck, 1:1,250 dilution). Horseradish peroxidase-conjugated secondary antibodies (Anti-Rabbit A6667, Anti Mouse A5906, Sigma Aldrich) were used to detect the bound primary antibody. Immune complexes were visualized by enhanced chemiluminescence (Amersham ECL Prime Western blotting reagent) following the manufacturer’s instructions.

### 2.9 Cell imaging experiment

The A2780 human ovarian cancer cells were seeded in Cellview cell culture dish (Greiner Bio one Ref. 627,870) at a confluence of 50% in a final volume of medium corresponding to 0.5 mL in each compartment. The day after, the cells were treated by adding the **E5-FITC** conjugated compound (50 μM) in two different ways: i) directly into the culture medium (in one cell culture dish) and ii) after transfection with SAINT-Protein (in the other cell culture dish). In the first case, cells were treated with 50 μM **E5-FITC** for 24 h; in the second case, for the transfection experiments, cells were treated with **E5-FITC** (50 μM) transfected into cells for 24 h using a previously optimised amount of SAINT-Protein. Complexes of **E5-FITC** (1.25 μL) with SAINT-Protein (8 μL) were prepared in sterile test tubes for the treatment in a Cellview cell culture dish without vortexing but gently pipetting three times. The volumetric ratio between the drug solution and the transfectant was maintained. The mixture was incubated for 5 min at room temperature, then filled to 250 μL with serum-free medium. The culture medium was aspirated from the cells, and the SAINT-Protein/drug complex in serum-free medium was gently added to the wells and incubated for 4 h (37°C; 5% CO2). After this time, a medium with 20% serum (250 uL) was added to reach the appropriate volume (500 uL). At the scheduled times, the tissue culture medium was removed from all compartments of the two cellview cell culture dishes, and the cells were washed two times with PBS and labelled with cellstain-Hoechst 33,342 solution-nucleus dye (blue) for 5 min. The cells were observed under a confocal microscope (Leica TCS SP8 with AOBS, WLL, STED 3X and FLIM), and different images were taken for each condition. Quantification of the level of green emission from the FITC probe, assumed to be representative of the amount of E5-FITC within cells, was done with the ImageJ software (https://imagej.net/ij/). For each of the experimental conditions, i.e., A2780 cells treated with 50 μM **E5-FITC** without and with 8 μL SAINT-Protein, from 6 to 9 images were analyzed by defining 5–8 regions of interest (ROIs) per image. For each ROI, the mean intensity of the blue and green emissions, respectively identifying the nuclei and the internalized **E5-FITC**. Thus, about 60 couples of readings were obtained per experimental condition (Saint/no Saint). Similarly, from ROIs defined in images of control experiments (cells + DMSO, cells + DMSO + Saint-Protein) the average levels of green emission were determined. This value, our green background, was subtracted from the values found with **E5-FITC** with and without Saint-Protein. Then, to normalize the FITC emission of each ROI to the corresponding blue emission, so as to account for experimental fluctuations from image to image, the green-to-blue intensity ratio was computed. Finally, these ratios were statistically analyzed to determine their mean values and standard deviations.

### 2.10 Statistical analyses

Results of the experiments are expressed as mean ± standard error of the mean (S.D.), unless otherwise indicated. A parametric two-tailed Student’s t-test was used to assess the statistical significance when two groups of unpaired normally distributed values were compared; when more than two groups were compared, parametric two-tailed analysis of variance (ANOVA) was applied. Data were analyzed with GraphPad Prism 8.0.1 (GraphPad Software). All mean differences were considered statistically significant if *p < 0.05; **p < 0.01; ***p < 0.001.

## 3 Results

### 3.1 The Ddis cytotoxicity is enhanced by plasma membrane drug-efflux transporter inhibitors

**FIGURE 2:**
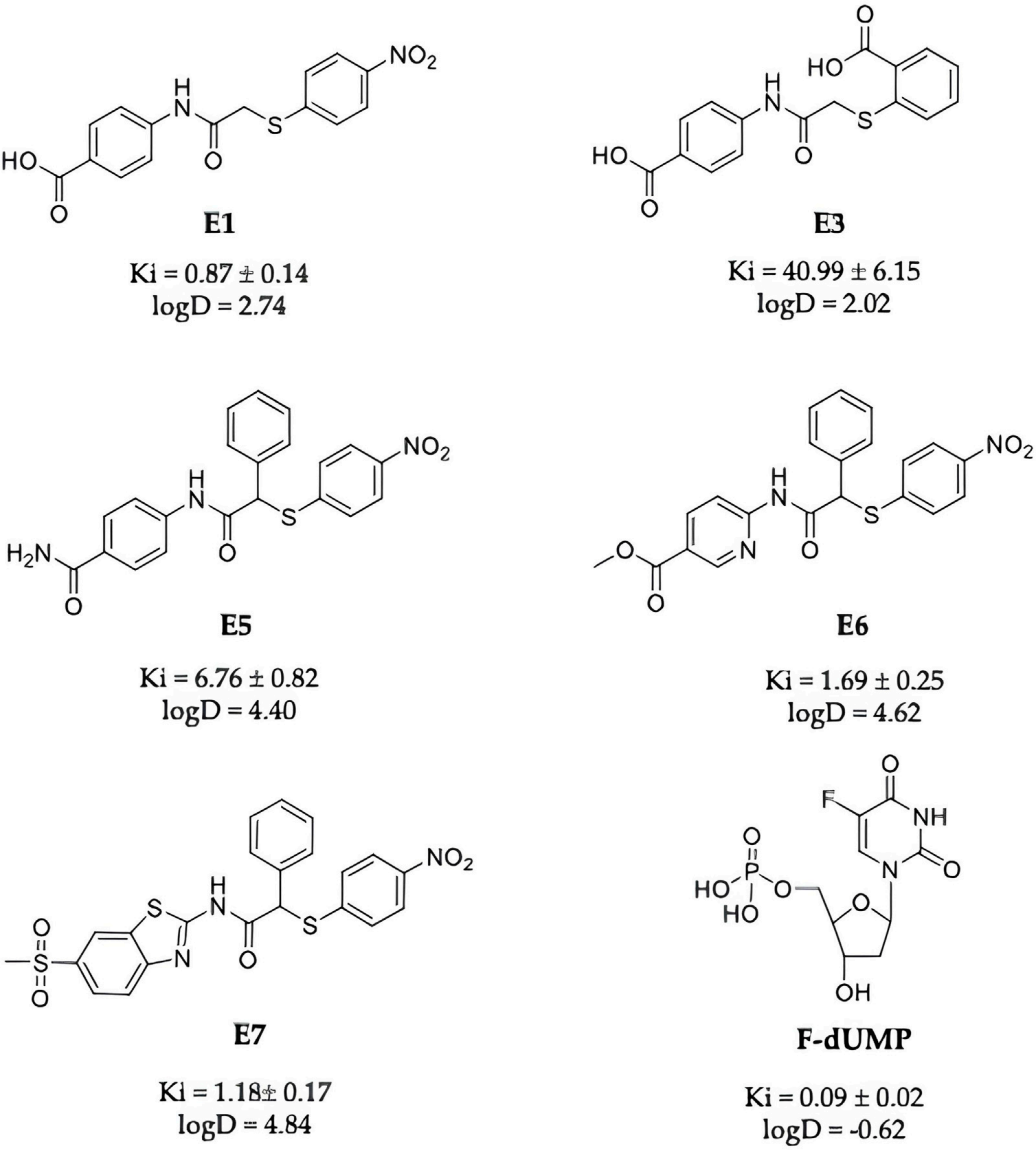
Chemical structures of **E1**, **E3**, **E5**, **E6**, **E7** and **FdUMP,** their IC_50_ against the *h*TS enzyme and logD values at pH 7.4.

Compounds **E1, E3, E5, E6, E7** (Figure 2) were selected among the most interesting compounds previously studied showing a broad range of enzyme inhibition and dissociative capacity, and cellular activity (Costantino et al., 2022). **E1** and **E3**, show IC50 values against recombinant hTS activity of 8 and 473 μM, respectively (Table 1), and can both destabilize the enzyme dimer (Costantino et al., 2022). They have logD’s of −0.18 and −3.23 [“Calculator Plugins were used for structure property prediction and calculation, Marvin 24.1.1, 2024, ChemAxon (http://www.chemaxon.com)”], and no observed cellular activity at 100 µM (Table 1). Compound **E3** was indeed interesting due to its high solubility (331.34 mg/mL, Supplementary Table S2) even if it had a low enzyme inhibition activity. It represents a control compound due to its very low activity on cancer cells and high water solubility. Instead, compounds **E5-E7** show IC50 values against recombinant *h*TS activity of 40, 10 and 7 μM and 10–70 µM against cancer cells (Costantino et al., 2022). As discussed in the methods section (Section 2.3) the inhibition mechanism of the Ddis compounds towards *h*TS needs careful evaluation depending on the capacity of the compounds to bind to the enzyme monomer. The Ddis are considered non-competitive inhibitors (see Section 2.3). **E7** is the most active compound among the Ddis tested both in ovarian (OC) and in colorectal cancer (CRC) cells, showing IC_50_ values in the same range as 5FU (Table 1). Specifically, it shows a similar IC50 value as 5FU on A2780 cells, and half the potency of 5FU on the A2780/CP, IGROV-1, HT29, and HCT116 cells. Compound **E6** shows a similar profile as **E7**, while **E5** is less effective on IGROV-1, HT29 and HCT116 cells. 5FU shows a similar effect on the 5 cell lines under study, while the action of the Ddis is more dependent on the cell type. Overall, the 5 compounds cover a broad range of chemical properties and biological activities.

**TABLE 1:** Biological activity of compounds **E1, E3, E5, E6, E7**.

Compound	IC50 (µM)^a^	Cellular IC_50_ values (µM ± S.D.)				
	Recombinant *h*TS	A2780	A2780/CP	IGROV-1	HT29	HCT116
**5-FU**	0.80 (as FdUMP)	8.5 ± 0.8	11.6 ± 1.2	16.9 ± 1	14.2 ± 3.0	13.5 ± 1.5
**E1**	8.2	>100	>100	>100	>100	>100
**E3**	470	>100	>100	>100	>100	>100
**E5**	40	28.8 ± 4	34.3 ± 5	63.9 ± 8	70.5 ± 1.6	61.8 ± 1.8
**E6**	10	19.5 ± 2	18.7 ± 1	39.1 ± 3	40.2 ± 5.2	36.5 ± 4.1
**E7**	7	10.1 ± 0.3	21.5 ± 3	37.4 ± 6	34.5 ± 3.0	35.5 ± 0.3

^a^IC_50_ values against the hTS recombinant enzyme.

IC_50_ values (50% cell growth inhibitory concentration; µM ± S.D.) obtained after 72 h-treatment of A2780, A2780/CP, IGROV-1 human ovarian cancer cell lines and HT29 and HCT116 human colorectal cancer cell lines.

A standard error on the data in triplicate is ±20%.

We observed a discrepancy between the capacity of Ddis to inhibit the *h*TS recombinant protein and their effectiveness in cancer cell growth inhibition, therefore we first wondered if the Ddis compounds could be substrates of the ABC (ATP-binding cassette) family efflux protein pumps. To this aim, we tested the efficacy of the combination of the Ddis in cancer cells with elacridar or tariquidar, ABC pump inhibitors. Compounds **E5** and **E7** were selected as representative compounds among the Ddis (Figure 2). These show different biological profiles whereas the cancer cell growth inhibition potency is 25–70 μM and 10–37 µM respectively; Ki ranges between 40 µM and 7 μM, respectively with similar logD higher than 4 because they are lipophilic compounds. **E5** contains an amidic function that is not expected to be hydrolyzed in cells. Considering the lipophilic nature of the compounds, we expected that these compounds could show affinity for the MDR protein, compete with elacridar and tariquidar, and be internalized.

Cells were pre-treated with 200 nM elacridar or 100 nM tariquidar for 2 h then Ddis were added, and the cell growth was observed for an additional 72 h. These concentrations were selected based on methods adopted in previous works for effective inhibitors of ABC transporters but scantly cytotoxic (Patel et al., 2011; Muz et al., 2017). **E5** and **E7** were also tested at concentrations lower than their IC50 values previously obtained (Costantino et al., 2022). The IC50 values of the two Ddis in A2780 cells are 28.8 ± 4 and 10.1 ± 0.3, respectively. In IGROV-1 cells were 69.9 ± 8 and 37.4 ± 6, respectively. In HT29 cells were 70.5 ± 1.6 and 34.5 ± 0.3, respectively. We have chosen two concentrations that cause, in A2780, IGROV-1, and HT29 cell lines, approximately 10% and 30% cell growth inhibition. The choice of concentrations much lower than the IC50 value is made to better highlight the enhanced effect of the combination compared to single agent administration.

**FIGURE 3:**
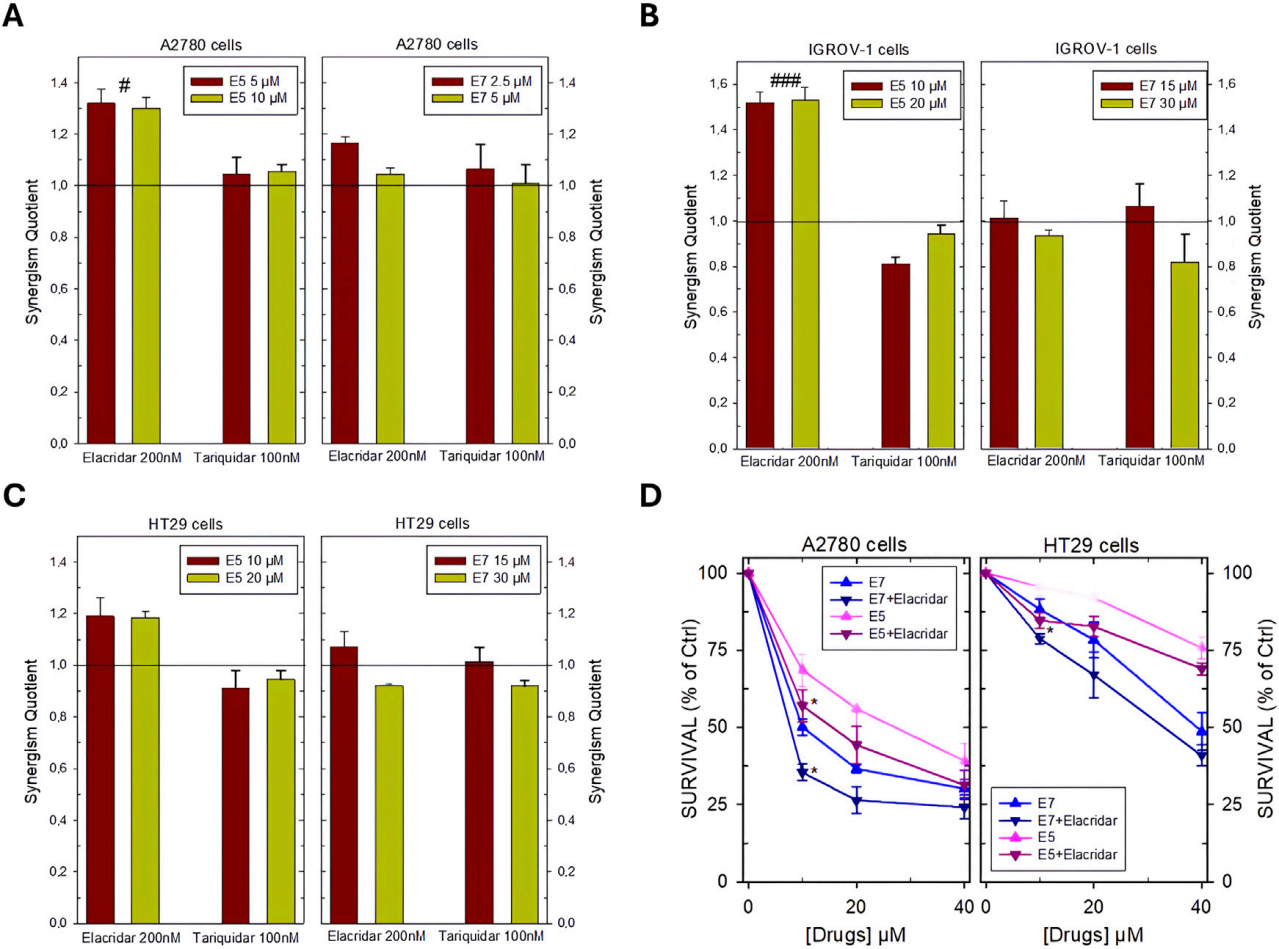
Synergism Quotient values after combining **E5** and **E7** with inhibitors of membrane ABC transporters in A2780 cells **(A)**, IGROV-1 cells **(B)** HT29 cells **(C)**. The bars represent the average of the duplicate cell counts from two separate experiments and indicate the values of the synergism quotient obtained by dividing the result of the inhibition of the drug combination by the sum of the inhibition of the single drug. SQ > 1 indicates synergism, while SQ < 1 or = 1 indicates an antagonistic or additive effect, respectively. **(D)**, effects of the combination of **E5** and **E7** with the inhibitor of the ABC membrane transporters elacridar on the dose-response curves of these Ddis in A2780 and HT29 cell lines. Cells were pre-treated with 200 nM elacridar for 2 h before adding the drugs for an additional 72 h. Data are the mean ± S.D. of three separate experiments performed in duplicate. *p* values were calculated with two-sided Student’s *t*-test and ANOVA multiple comparisons. ^#^ *p* < 0.05; ^###^ *p* < 0.001, comparing samples treated with elacridar or tariquidar. *p < 0.05; versus samples without pump blockers.

Data were obtained to allow the analysis of the interaction between the two inhibitors, ABC transporter inhibitors, and Ddis, through the synergism quotient (SQ) (Figures 3A–C). The data obtained show favorable interaction effects, producing synergistic cell killing in most of the combinations tested in all cell lines in the presence of elacridar. Instead, an additivity was observed in the combination of the Ddis with tariquidar, with the exception in IGROV-1 cells, in which some SQ values indicate slight antagonism (Figure 3B).

Dose-response curves were then obtained for the combinations that proved more favorable, i.e., Ddis plus elacridar in A2780 and HT29 cell lines showing SQ from 1.2 to 1.35. Cells were pre-treated with 200 nM elacridar for 2 h before adding increasing concentrations of **E5** or **E7** for an additional 72 h and then processed for the evaluation of cell growth inhibition. In both cell lines, the combination of **E5** or **E7** plus elacridar showed a greater cellular inhibition at the lower concentrations, 10 and 20 µM, lowered the survival rates by about 10%–15%, compared to the administration with the Ddis alone (Figure 3D). In the A2780 cells, the IC_50_ values were decreased by elacridar co-treatment from 10.1 ± 0.3 to 7.7 ± 0.5 µM and from 28.8 ± 4 to 15.4 ± 2 for **E7** and **E5**, respectively. In HT29 cells, for **E7** was determined an IC_50_ reduction from 34.5 ± 3–30.3 µM.

The results in Figure 3 indicate that the Ddis compounds are substrates of the cellular efflux protein pumps; their effectiveness can be increased by enhancing their intracellular accumulation by blocking the extrusion proteins.

### 3.2 Facilitating the uptake and effectiveness of the Ddis compounds by transfection

**FIGURE 4:**
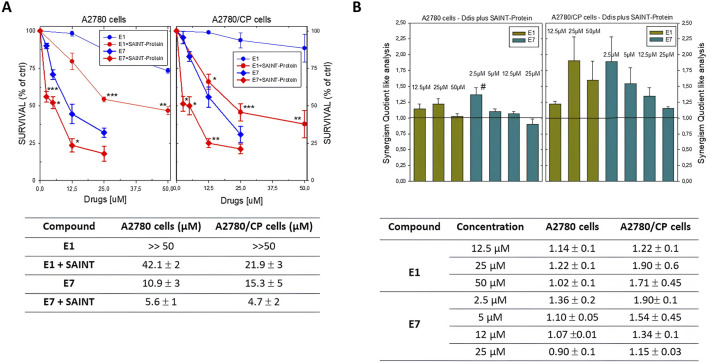
**(A)** Dose-response curves of Ddis compounds in the absence or presence of the SAINT-Protein transfection system in A2780 and A2780/CP cell lines. Bottom, Table of the IC_50_ (50% cell growth inhibitory concentration; µM ± S.D) values for the Ddis compounds **E1** and **E7** with or without SAINT-Protein after 72 hour-treatment of A2780 and A2780/CP human OC cell lines. **(B)** Histogram of the synergism quotient like analysis to evaluate increased efficacy obtained by dividing the result of the inhibition of the combination of compounds with SAINT-Protein by the sum of the inhibition of the single component. A quotient> 1 indicates synergism, while a quotient <1 or = 1 indicates an antagonistic or additive effect, respectively. Bottom, Table of the SQ values. Data indicate mean values and standard deviation of at least two biological repeats performed in duplicate. *p* values were calculated with two-sided Student’s *t*-test and ANOVA multiple comparison. *p < 0.05; **p < 0.01; ***p < 0.001.; versus not transfected samples. #*p* < 0.05, versus treatments with other concentrations of **E7** plus SAINT-Protein.

We tested the effects of **E1**, **E3**, and **E5**-**E7** compounds administered in association with the non-liposomal transfection reagent SAINT-Protein. The SAINT-Protein does not form micelles but, by fusing with the membrane’s phospholipid bilayer, releases the agent inside, without involving the endosome. It is generally used to deliver peptides, and not for small molecules. Based on the experience gained using SAINT-Protein (previously SAINT-PhD) for the transfection of octapeptides into tumor cells, we have adapted and optimized this method to deliver the Ddis small molecules as reported in the methods section. The optimization consisted in finding the right ratio between drug solutions at the appropriate concentrations and the volume of SAINT-Protein that retained a <5%. cellular cytotoxic effect. After several tests on the four considered cell lines, the most appropriate volume of SAINT-Protein, suitable for this purpose, was 1/3 or 1/4 the volume recommended by the manufacturer and usually used by us to convey peptides. The volumes used were 8 uL and 20 µL, for the 24-well plate and 6-well plate experiments, respectively. We performed confocal fluorescence microscopy imaging of living cells to demonstrate the increase in cellular uptake of our compounds caused by the SAINT-Protein transfection agent. For these experiments, we employed compound E5 conjugated with the green-emitting FITC fluorescent dye (E5-FITC) and the A2780 cells (more details given in paragraph 2.9 of Materials and Methods). The level of the green signal was assumed to reflect the amount of E5-FITC internalized. We determined its average value in 60 regions of interest (ROIs) of the acquired images for each of two experimental conditions, i.e., A2780 cells + E5-FITC with/without SAINT-Protein. We found that the average value in the presence of the transfection agent, 0.15 ± 0.06, was nine times larger than the average value found in its absence, 0.017 ± 0.008 (representative confocal microscopy images are provided in Supplementary Figure S4). Increasing concentrations of the compounds were added while always maintaining the same volumetric ratio of 4 between the drug solutions and the transfectant for both cytotoxic and cell cycle experiments. SAINT-Protein transfection reagent formulation consists of 2 principal lipid components, the pyridinium cationic amphiphile Saint and the phospholipid DOPE (1,2-dioleoyl-*sn*-glycero-3-phosphoethanolamine), which is neutral at physiological pH and is a well-known endosomolytic reagent. This condition appears suitable to convey our compounds, especially those which are negatively charged such as **E1** and **E3,** at the pH of the culture medium. Compounds **E1** and **E7** were studied first, to evaluate their capacity to improve their respective *in vitro* IC50 when delivered using SAINT-Protein. **E1** alone cannot inhibit cancer cell growth even at the highest concentration, while **E7** is a good inhibitor that can reduce cancer cell growth like 5FU in some cases (Table 1). As shown by the dose-response curves for compounds **E1** and **E7** (Figure 4A) and the synergy analysis (Figure 4B), the administration of the Ddis compounds in the presence of SAINT-Protein enhances their effectiveness with respect to exposure in the absence of the transfectant.

Although the compound **E1** has been shown to inhibit the activity of the recombinant enzyme (IC50 = 8.2 μM), when administered to the cells individually it shows very scant activity in both cisplatin-sensitive and -resistant OC cell lines (IC50 > 100 μM, Table 1; Figure 4A). In fact, to obtain inhibitory effects on 50% of the cell population, the compound required concentration is much higher than that of the Ddis **E7**. The data collected show how the combination with the transfection reagent SAINT-Protein, favors the achievement of cellular inhibition at 50% for doses of **E1** equal to less than half of those of its IC50 value, if not transfected. Its IC50 decreases from >100 μM to 20–40 μM. We defined the system SAINT-Protein combined with the Ddis, as a synergistic system in which a synergism quotient (SQ) study could apply. The results obtained for the evaluation of the SQ analysis reveal a marked enhancement of cytotoxicity after transfection with a quotient >1, particularly at lower concentrations (Figure 4B; Supplementary Table S1). The SQ values fluctuate between 1.14 and 1.02 for A2780 cells and 1.22 and 1.7 for A2780/CP cells, respectively. This result indicates the SAINT-Protein as a promising delivery system in favouring the entry of the compounds into the cell, thus allowing an increase in the efficacy even of less active compounds showing a poor cellular uptake (**E1**). **E7** shows an increase in cytotoxicity presenting an IC50 decrease of about 50% (from 10.9 to 5.6 μM) in association with SAINT-Protein, the SQ values fluctuate decreasing between 1.36 and 0.9 for A2780 cells and 1.9 and 1.15 for A2780/CP cells, respectively. Noteworthy, the enhanced effect is more evident in the resistant than in the sensitive cells (Figure 4B; Supplementary Table S1).

**FIGURE 5:**
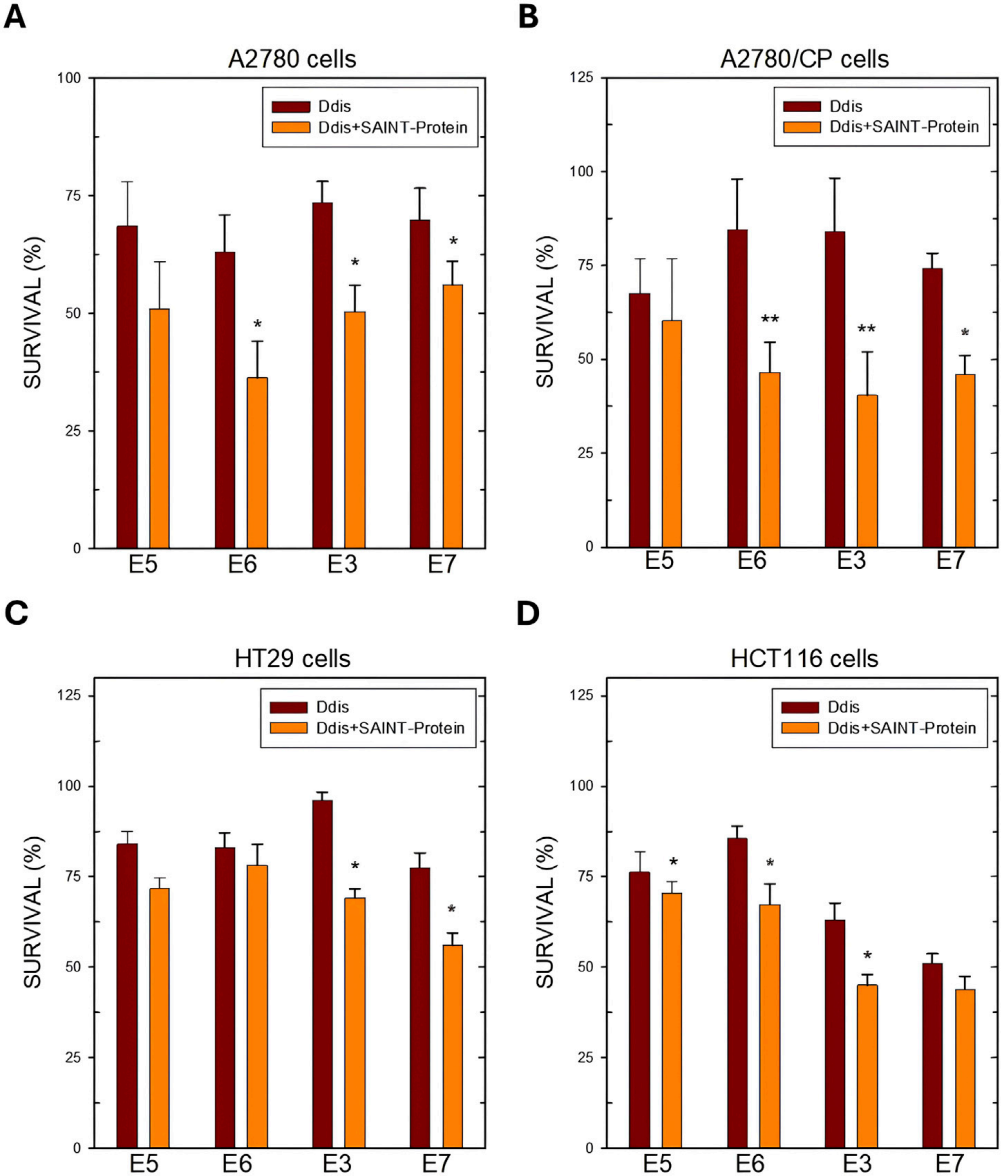
Cell survival after 72 h of treatment with the indicated Ddis drugs in the absence or presence of the SAINT-Protein transfection system in OC A2780 **(A)** and A2780/CP cell lines **(B)** and in HT29 **(C)** and HCT116 **(D)** CRC cell lines. Data indicate mean values and standard deviation of at least two biological repeats performed in duplicate. *p* values were calculated with a two-sided Student’s *t*-test, comparing transfected and non-transfected samples. *p < 0.05; **p < 0.01; versus non transfected samples.

Other Ddis compounds show a higher effect when delivered with SAINT-Protein, but their effectiveness is more cell-type-dependent (Figure 5). As shown in Figures 5A, B, the Ddis compounds, transfected into A2780 and A2780/CP cell lines, at concentrations lower than the respective IC50 values (usually the IC25%), significantly increased cell killing from approximately 15%–30%, particularly **E3** and **E6** (Figure 5A). Only the action of compound **E5** was not significantly enhanced by the transfection; this is more evident in the resistant A2780/CP cells.

In HT29 CRC cells, only **E3** and **E7** are more favoured by the presence of SAINT-Protein, while for **E5** and **E6** no significant effect was observed (Figure 5C). The enhanced effect on cell growth inhibition of the Ddis **E3** and **E7** following transfection correlated with a higher *h*TS protein reduction, particularly evident for the transfected **E3** (Figure 7A), due to *h*TS protein degradation, a well-known downstream effect predicted by the disruption of *h*TS dimers by the Ddis (Costantino et al., 2022). Indeed, HCT116, which has a different morphology and growth modality, responds more sensitively to Ddis compounds when they are transfected into cells by SAINT-Protein, producing significantly different results (Figure 5D). In conclusion, the Ddis **E1** that inhibits the *h*TS recombinant enzyme *in vitro* but not the cell growth due to their reduced uptake across the plasma membrane benefits considerably from being conveyed with this delivery system. **E3** shows an overall limited efficacy due to its much lower affinity for the target (approximately 20% inhibition at 100 μM), nevertheless, the enhancement in the efficacy due to the increased intracellular accumulation, can be observed also in this case. The cell inhibition potency of the compound increased when these are delivered in combination with the SAINT-Protein and the observed efficacy parallels the IC_50_ against the recombinant *h*TS protein. The observed trend is consistent with the ability of Ddis to engage *h*TS intracellularly with low off-target effects. This also shows that the delivery system does not alter the compound intracellular targeting.

#### 3.2.1 Ddis-induced cell cycle modulation

In previous studies, we observed that **E7** showed an apoptotic effect via caspase 3 activation (Costantino et al., 2022). Based on those results, we investigated whether the effects on cell growth of **E7** derivatives in combination with SAINT-Protein were related to cell cycle modulation by flow cytometry analysis. As shown in Figure 4, **E3** action was significantly enhanced by transfection in all tested cell lines. We performed cell cycle analysis using **E3**, the Ddis with the lowest logD value, in comparison with **E7**, with the highest logD value (Supplementary Table S2), in two cell lines, A2780/CP cells, and the HT29 cells. Both cancer cell lines are resistant to folate cycle conventional inhibitors both inductively and innately respectively.

**FIGURE 6:**
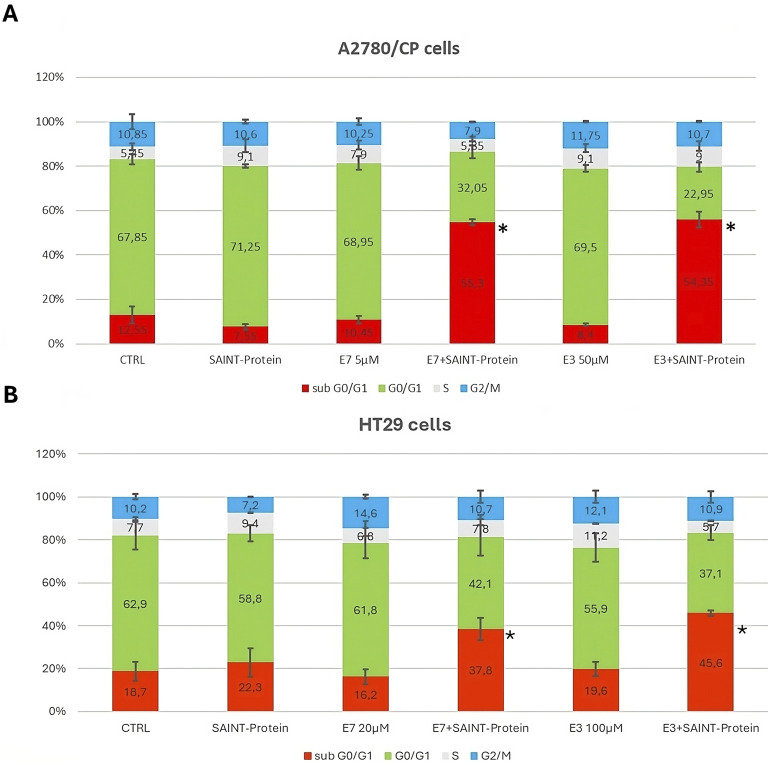
Effect of the combination of the Ddis drugs with SAINT-Protein for 72 h on the distribution of cell cycle phases in the A2780/CP OC cells and HT29 CRC cells by flow cytometric analysis of DNA content with PI staining. The changes in cell distribution at different stages of the cell cycle in A2780/CP cells **(A)** and HT29 cells **(B)** were observed when cells were treated with **E3** and **E7** with or without SAINT-Protein. Inserted numbers indicate the % of cells in the different phases of the cell cycle. Data indicate mean values and standard deviation of at least two biological repeats performed in duplicate. *p* values were calculated with a two-sided Student’s *t*-test, comparing transfected and non-transfected samples. *p ≤ 0.05, **p < 0.01, in comparison to non-transfected drug samples.

Figure 6A shows that the A2780/CP cells untreated, taken as CTRL, present a diploid distribution typical of fast-proliferating healthy cells. As shown in Figure 6A, both Ddis compounds tested in single administration at 5 and 50 μM, respectively, i.e., concentrations much lower than the respective IC50s (Table 1) did not cause significant cell cycle perturbation, while in combination with SAINT-Protein, they determined a significant accumulation of cell population in the sub-G1 phase, (*p* < 0.001). The sub-G1 cell accumulation was accompanied by a corresponding decrease in cell distribution in the G0/G1 cell cycle phase.

Figure 6B reports the effects on HT29 cell cycle modulation of the Ddis compounds in association with the transfection reagent. It can be observed an uneven distribution of the various phases of the cell cycle, probably due to the characteristics of this tumor cell line. Nevertheless, the increased number of cells in the sub-G1 phase following the Ddis plus SAINT-Protein combination is also evident for these cells, for both combinations, albeit quantitatively lower (45%) with respect to A2780/CP (57%).

Owing to the scant sensitivity of HCT116 cells to **E5** and **E6** (Table 1), these CRC cells were transfected only with **E7**, and with **5-FU** for comparison. **5-FU** plus SAINT-Protein causes a reduction of cancer cells in the G0/G1 phase and a corresponding increase in the G2/M and sub-G0/G1 phases of the cell cycle. These results were pronounced at 72 h for both **5FU** and **E7,** with about 40% and 60% of sub-G0/G1, respectively. Conversely, in OC A2780 cells, **5FU** did not benefit from SAINT-Protein transfection, while transfected **E7** significantly increased the percentage of cells in the sub-G0/G1 phase. Noteworthy, according to cell growth inhibition activity depicted in Figure 4A, B, the Ddis E1, poorly active when administered alone at this concentration, after transfection, greatly blocked cells in this phase and caused a corresponding reduction of cellular accumulation in the G0/G1 cell cycle phase (see Supplementary Figures S1, S2). The effect was significant (*p* < 0.05).

#### 3.2.2 Effect of compound **E3** on *h*TS protein levels following HT29 cancer cells treatment

**FIGURE 7:**
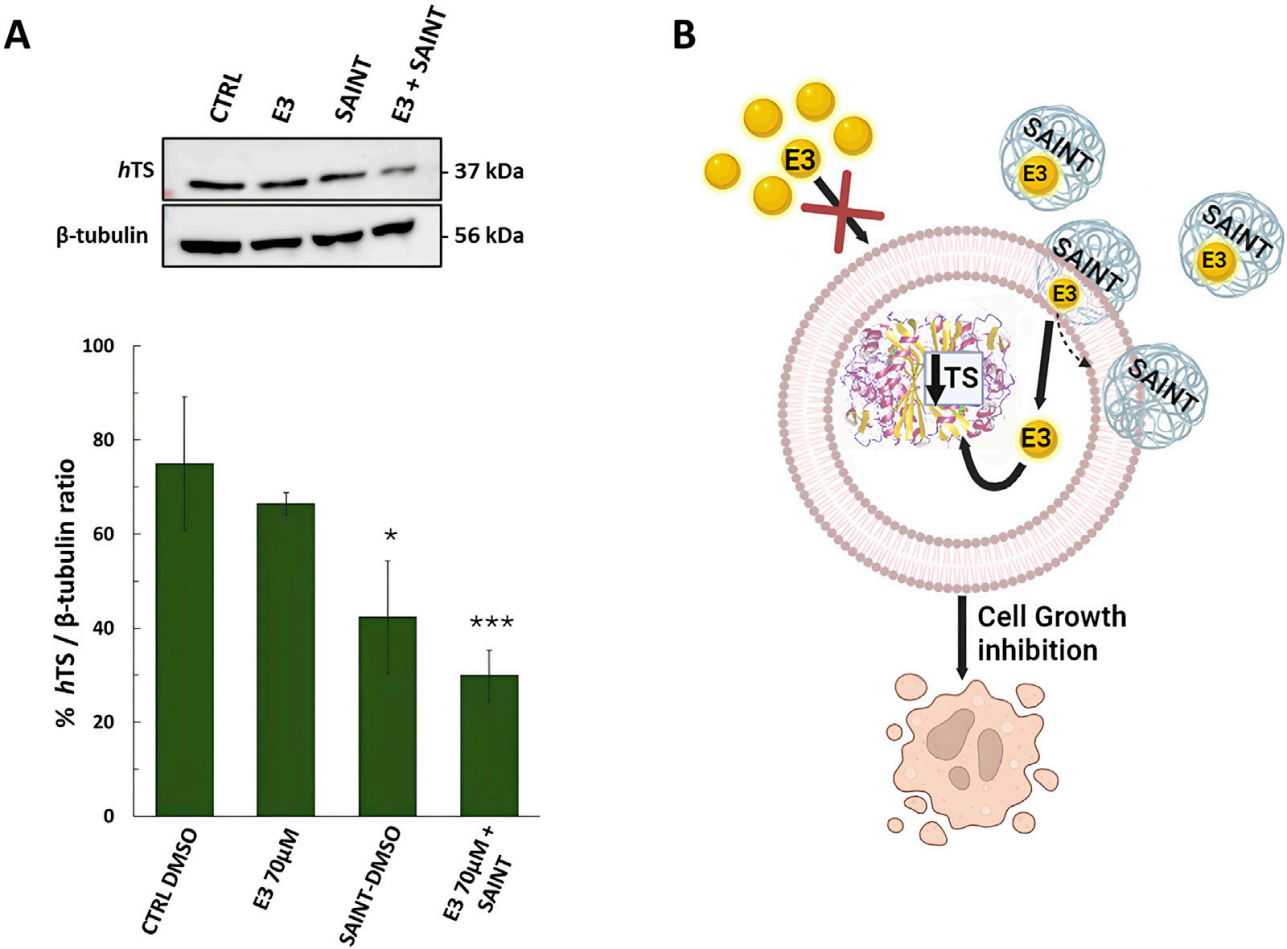
Effects of compound **E3** on the expression levels of thymidylate synthase (*h*TS) protein in HT29 colon cancer cell line, after 48 h of treatment. **(A)** Representative Western blot and quantification histogram for *h*TS expression levels after **E3** was administered alone or with SAINT™-Protein. β-Tubulin has been used as a housekeeping protein. The bands obtained by immunoblot were analyzed by ImageJ (Java-based image-processing and analysis software). **(B)** Cartoon illustrating the expected mechanism of transfection of the SAINT-Protein (sphere pale blue) to internalize the drug (yellow sphere) into the cell. Once in the cell, the drug is free to interact with *h*TS and shift the equilibrium towards the monomer followed by its degradation. This causes the *h*TS level to decrease and the cell growth inhibition. Cartoon created using BioRender™ free trial plan. Data indicate mean values and standard deviation of at least two biological repeats performed in duplicate. *p* values were calculated with a two-sided Student’s *t*-test, comparing transfected and non-transfected samples. *p < 0.05; ***p < 0.001 vs. E3.

Since *h*TS is an essential and abundant protein in CRC cell lines, and since it is also the target protein of our compounds (Ddis), we set out to define whether the **E3** compound (with the lowest logD value), internalized by using the SAINT-Protein delivery system, regulates the endogenous *h*TS protein expression by performing a Western blotting assay. As expected from our previous report (Costantino et al., 2022), the cellular *h*TS level decrease is an indicator of the Ddis dissociative action. Here we showed that the level of *h*TS protein was markedly and significantly reduced (up to 2-fold) upon **E3** treatment (70 µM for 48 h) in HT29 cells by using the SAINT-Protein delivery system (Figures 7A, B). Noteworthy, the *h*TS protein reduction is observed only when **E3** is internalized into the cells by using the delivery system, but not if **E3** is used alone (Figures 7A, B). Those results agreed with the enhanced effect on cytotoxicity and cell cycle perturbations of **E3** internalized into the cells by using the SAINT-Protein with respect to **E3** alone. The *h*TS protein reduction observed after internalized **E3** by the delivery system confirms its action to be based on the ability to disrupt the *h*TS target protein dimer as previously demonstrated for other Ddis.

## 4 Discussion

Overcoming biological barriers to achieving therapeutic proof-of-concept for drugs’ optimal pharmacokinetic and pharmacodynamic is of paramount relevance in the perspective of drug development. As a general concept, drug delivery systems have emerged as a modern approach to improving drug performance. They enhance the pharmacokinetic properties of the drugs, ensure their delivery to the tumor, prevent toxicity to healthy tissues, increase drug concentration at the tumor site and therefore potentiate their anticancer activity while mitigating their associated side effects. In combination therapy, they can effectively carry several drugs with dissimilar pharmacokinetics profiles and maintain the optimized synergic drug ratio until they reach the target cancer cells. Recent studies in efflux pumps expressing cancer show convincing effects of those inhibitors (Zaidi et al., 2017). Novel drug delivery approaches are investigated for potential utility in CRC therapeutics including systems based on microspheres, micelles, nanoparticles, liposomes, microsponge, and microemulsions providing enhanced solubility and bioavailability of poorly water-soluble drugs used in CRC (Singh et al., 2022a). There are various liposome-based drugs available in the markets approved by the US Food and Drugs Administration (FDA) to treat various tumors, including leukemia, breast cancer, colorectal cancer, lung cancer and brain cancer (Singh et al., 2022b). Among other delivery systems, it is currently, proposed the GMP-grade SAINT lipids that are being used in a clinical trial as reported in (Roeven et al., 2015) and in several applications of animal models (Endmann et al., 2014). In the context of *h*TS inhibition and drug resistance studies in the presented work, we directed our interest in the SAINT-Protein delivery system, a variant of the mentioned SAINT-lipid, applied to protein transfection in cancer cells. Its potential application in translational studies and, in perspective, in the early clinical oncology setting motivated our novel application to the small molecules. In this context, we have studied novel anticancer leads showing previously unexplored mechanism of action, the mentioned dimer disrupter TS inhibitors, namely, Ddis (Costantino et al., 2022).

The disrupters of the *h*TS dimer administered to OC and CRC cell lines in this work have previously been shown to inhibit enzymatic activity and markedly trigger its proteasomal degradation, resulting in cancer cell growth inhibition both in cultured cells and in mouse models and, unlike traditional hTS inhibitors, not accompanied by over-expression of this critical enzyme for DNA synthesis and thus by the consequent resistance to further treatments (Costantino et al., 2022). Therefore, Ddis represent a new approach to avoid anti-TS drug resistance and are promising tools against cell growth in different tumor types.

While some of these drugs showed low micromolar inhibition of hTS and protein dimer disrupting activity (Costantino et al., 2022), they did not cause a measurable inhibition of cancer cell growth, possibly because of poor cellular uptake and/or fast extrusion. Among them, both compounds **E1** and the **E3** (highly soluble, low potency compound) contain at least one anionic group at pH 7 that can prevent their internalization in cancer cells, due to electrostatic repulsion with the membrane phospholipidic negative charge. **E1** and **E3** (logD −0.18 and −3.23) also show a logD much lower than **E5**-**E7** (logD 4.47 and 4.84) and are therefore more suitable to interact with the outer membrane and be internalized through it. In order to verify whether the less cytotoxic Ddis compounds that can inhibit the recombinant enzyme, such as **E1** and **E3** could improve their efficacy in inhibiting cancer cell growth, we increased their intracellular concentration. On the other hand, we also wanted to see if increasing **E5**-**E7** intracellular concentration their efficacy could improve. To this end, we first tested the two compounds **E5** and **E7** in combination with two different drug extrusion pump inhibitors, tariquidar and elacridar (pump blockers), to determine which of them benefited most from the combined treatment. Both pump blockers exert high inhibition against the pump, and in our conditions, they affect the action of the most potent Ddis, **E7**, with similar additivity.

We noticed differences in the action of the less active **E5**, whose cytotoxicity was synergized by elacridar in all cell lines tested. The reason for this different behaviour could depend on a different interaction of **E5** and elacridar with the extrusion proteins that could be more specific than with **E5** and tariquidar, resulting in a possible increased accumulation of **E5** in all cells and enhanced cell killing. Here, we found that our TS disrupters (Ddis), featuring a different molecular mechanism with respect to known anti-TS drugs, can be combined with inhibitors of drug efflux pumps. Our findings suggest that the target specificity and low toxicity of the Ddis can be explored *in vivo* in combination with clinically approved pump efflux inhibitors (Opdam et al., 2012) to start a promising translational study.

Recently, we reported the synergistic anti-tumor effects of an anti-TS peptide in association with some chemotherapeutics and delivered into cells both via the SAINT-PhD delivery system and via pH-sensitive pegylated liposomes (Edwards et al., 2015). Guidance from the National Institute for Health and Care Excellence (NICE) recommended pegylated liposomal doxorubicin hydrochloride (PLDH), alone or together with platinum, for the treatment of recurrent ovarian cancer (Edwards et al., 2015). In this study, we tested the effects of some Ddis compounds administered in association with the non-liposomal transfection reagent SAINT-Protein.

Compounds **E1** and **E3**, which are inactive as compounds *per se*, showed cytotoxic activity when associated with the SAINT-Protein. The Ddis accumulate and increase their intracellular concentrations, which cannot be achieved without this delivery system, leading to greater cell death and a decrease in *h*TS protein levels by promoting *h*TS protein degradation (Costantino et al., 2022). It is worth noting that the large decrease of **E1** ’s IC50 (increased efficacy of the cancer cell growth inhibition) when it is combined with the SAINT-Protein, was much higher than in the case of **E7**. A similar effect was observed for **E3.** This can be due to a more favourable interaction of the negatively charged carboxylic group of **E1** and **E3** with the cationic chemical groups of the SAINT systems. The ionic interaction can increase the stability of the complex formed by the compounds to be delivered and the non-liposomal system. Therefore, as a general concept, the presence of negatively charged chemical groups in the compounds to be delivered can increase the ionic interactions, the stability of the complex, and ultimately the efficacy of the delivery. The increased intracellular amount explains the enhanced observed effects on cell cycle perturbation such as an accumulation of hypodiploid cells in sub-G0/G1 phase, the induction of cleaved caspase-3, and the reduction of *h*TS protein levels following the administration of compound **E3**. Thus, among all the delivery systems available, the SAINT delivery system can be a promising delivery vehicle for drugs for *in vivo* applications, including oncology clinical settings.

## 5 Conclusion

In this report we demonstrate that the Ddis compounds can be made more effective by enhancing their intracellular accumulation, either by acting to increase their uptake or reducing their efflux from cells. The increased drug efficacy following the combination of the Ddis with MDR efflux pump inhibitors indicates that the accumulation of these compounds is affected by the presence of efflux pumps. In addition, the Ddis compounds also benefit from combination with the transfection agent SAINT-Protein, whose role as a delivery system has been successfully exploited as a facilitator in overcoming biological barriers. This action appears especially useful for those compounds that, despite inhibiting the activity of the recombinant *h*TS enzyme *in vitro*, show their cytotoxic efficacy limited by the obstacle of biological membranes. In this pilot study, we highlight that the two strategies to overcome multidrug resistance (MDR), through efflux pump inhibition, and by achieving a higher dose of drugs into the cells through the SAINT-Protein, avenue the delivery of these compounds *in vivo*, including oncology clinical settings. The translational study will allow their potential to overcome 5FU drug resistance in CRC patients to be exploited.

## Data Availability

The original contributions presented in the study are included in the article/Supplementary Material, further inquiries can be directed to the corresponding authors.
