# Supplementary material for: Enhanced anticancer effect of thymidylate synthase dimer disrupters by promoting intracellular accumulation
Source: Front Pharmacol. 2024 Nov 11;15:1477318. doi: 10.3389/fphar.2024.1477318 (PMC11602703; doi:10.3389/fphar.2024.1477318)

Original images for Blots/Gels

Figure 7

EXP 1

ZRB2019
Sigma-Aldrich
Anti-TYMS Antibody, clone 1E20 ZooMAb®
Rabbit Monoclonal
https://www.sigmaaldrich.com/IT/it/product/sigma/zrb2019

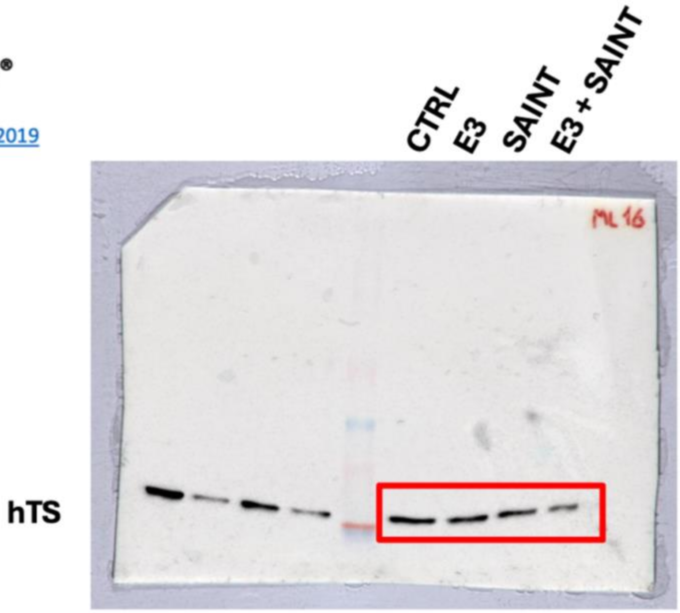

Anti-β-Tubulin Antibody, clone AA2
https://www.sigmaaldrich.com/IT/it/product/mm/05661i?utm_source=google&utm_medium=cpc&utm_campaign=20856413180&utm_content=162338192051&gclid=Cj0KCQiAh8OtBhCQARIsAIkWb69zh7__JvkWPu77yumFsnxLU-64wLOAM2eFlg0CiBCJvsnFaqpkEgAaAs9bEALw_wcB

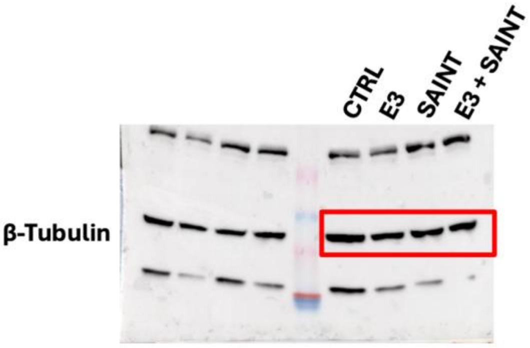

EXP 2

ZRB2019
Sigma-Aldrich
Anti-TYMS Antibody, clone 1E20 ZooMAb®
Rabbit Monoclonal
https://www.sigmaaldrich.com/IT/it/product/sigma/zrb2019

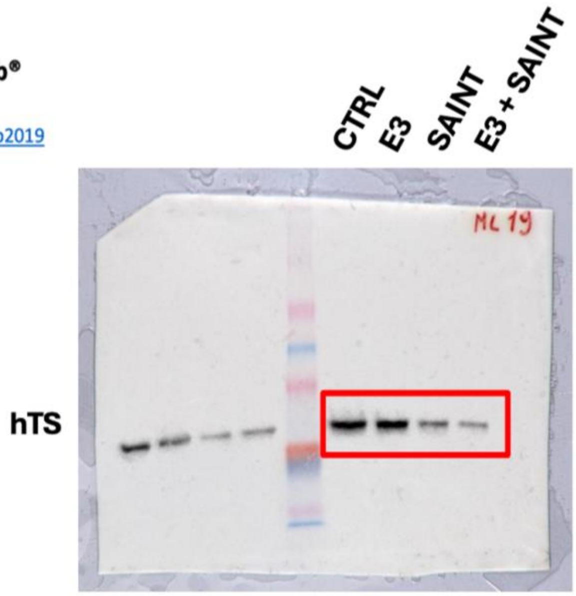

Anti-β-Tubulin Antibody, clone AA2

https://www.sigmaaldrich.com/IT/it/product/mm/
05661i?utm_source=google&utm_medium=cpc&ut
m_campaign=20856413180&utm_content=162338
192051&gclid=Cj0KCQiAh8OtBhCQARIsAIkWb69zh7
_JvkWPu77yumFsnxLU-
64wLOAM2eFlg0CiBCJvsnFaqpkEgAaAs9bEALw_wc
B

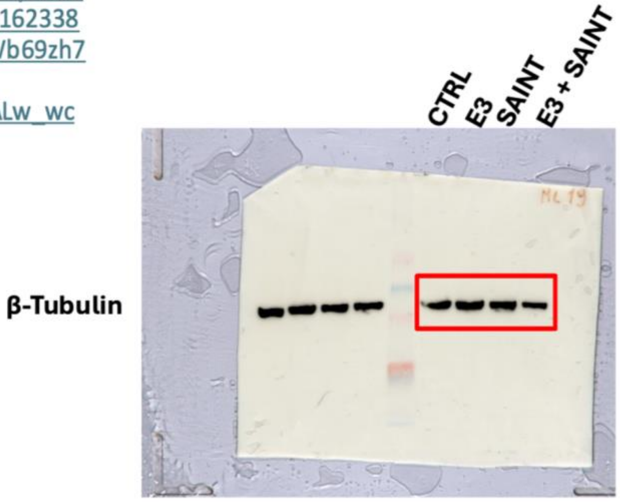

Supplement: Supplementary file 1 [file DataSheet2.pdf]
