# Supplementary material for: Enhanced anticancer effect of thymidylate synthase dimer disrupters by promoting intracellular accumulation
Source: Front Pharmacol. 2024 Nov 11;15:1477318. doi: 10.3389/fphar.2024.1477318 (PMC11602703; doi:10.3389/fphar.2024.1477318)
Supplement: Supplementary file 2 [file DataSheet1.pdf]

Supplementary Material

1 Supplementary Data

Enhanced Anticancer Effect of Thymidylate Synthase Dimer Disrupters Promoting Intracellular Accumulation

Gaetano Marverti[1*], Maria Gaetana Moschella[2,4], Alice Belardo[1], Michele Lamesta[4,5], Giada Mercanile[1], Lorenzo Tagliazucchi[2,4], Daniele Aiello[2], Alberto Venturelli[2], Davide Illuminati [6], Remo Guerrini [6], Lorena Losi[2], Glauco Ponterini[2], Maria Paola Costi[2*], Domenico D'Arca[1*]

[1]Department of Biomedical, Metabolic and Neural Sciences, University of Modena and Reggio Emilia, 41125 Modena, Italy

[2]Department of Life Sciences, University of Modena and Reggio Emilia, 41125 Modena, Italy.

[3]Department of Medical and Surgical Sciences for Children & Adults, University of Modena and Reggio Emilia, Modena, Italy.

[4]Clinical and Experimental Medicine PhD Program, University of Modena and Reggio Emilia, Modena, Italy.

[5] Doctoral course in Precision Medicine (course of national interest), University of Palermo, Italy.

[6] Department of Chemical and Pharmaceutical Science, University of Ferrara, Ferrara, Italy.

*****Correspondence: gaetano.marverti@unimore.it (G.M.); mariapaola.costi@unimore.it (M.P.C.); domenico.darca@unimore.it (D.D.)

Table of contents

**Scheme S1**	Synthetic procedure for compounds E1 and E3	Page 2
**Scheme S2**	Synthetic procedure for compound E7	Page 4
**Table S1**	SQ values	Page 5
**Table S2**	E1, E3, E5-E7 chemo-physical properties calculation	Page 6

**Figure S1**	Cell cycle phase distribution analysis of A2780 Cells	Page 8
**Figure S2**	Cell cycle phase distribution analysis of HCT116 Cells	Page 9
**Scheme S3**	hTS intact Mass Spectrometry protein analysis	Page 9
**Table S3**	UHPLC Gradient for hTS sample analysis	Page 10
**Figure S3**	UHPLC-HRMS analysis of intact hTS	Page 10
**Figure S4**	Cell imaging experiment	Page 12
**References**		Page 13

Synthetic procedure for compounds E1 and E3

Synthesis and characterization of E1, E3 compounds

Unless otherwise specified, all reagents were obtained from commercial sources (Sigma Aldrich, Merck, Fluorochem, BLD, ABCR) and used without further purification. The reactions were monitored by thin layer chromatography (TLC) with 0.20 mm EMD/millipore silica plates (60-F254), using UV light (λ =254 nm) as developing agent, Cerium Ammonium Molybdate (CAM), followed by a short heating with a heat gun. The ^{1}H and ^{13}C NMR spectra were recorded with a Bruker DPX-400 spectrometer or with a Bruker FT-NMR Avance III HD 600 MHz. The chemical shifts are reported on a δ scale with solvent residual signals as internal references (δH = 7.26, δC = 77.16 CDCl3; δH = 2.50, δC = 39.52 DMSO–d6). Two-dimensional NMR techniques (COSY, HSQC, HMBC) were employed for signal assignment in the ^{1}H and ^{13}C spectra. The chemical shifts are expressed in ppm (s = singlet, d = doublet, t = triplet, q = quartet, dd = double doublet, m = multiplet, br = broadened signal); the coupling constants (J) are expressed in Hz. High-resolution mass spectra were obtained using MeOH as solvent and Thermo Fisher HPLC-MS UltiMate 3000 mass spectrometer, with hybrid Q Exactive quadrupole – HESI-II electron spray orbitrap mass analyzer. Purity was determined using the HPLC area method. A HPLC-UV/Vis (Agilent Infinity II). Chromatographic separations were carried out on a RP Kinetex 2.6 μm Biphenyl 100 Å maintained at 30°C. The separation was performed under gradient conditions using solvent A t_0 95% - 5% over 22 min at a flowrate of 1 mL/min. Eluent A (H_2O +0.1% Formic acid), eluent B (ACN +0.1% Formic acid). The separation was monitored using wavelengths of 220 and 254 nm. Compounds **E1**, **E3** and **E7** were synthetized using modified synthetic procedures with respect to the ones reported in ref. [1]. Compounds **E5** and **E6** have been synthetized as reported in ref. [1].

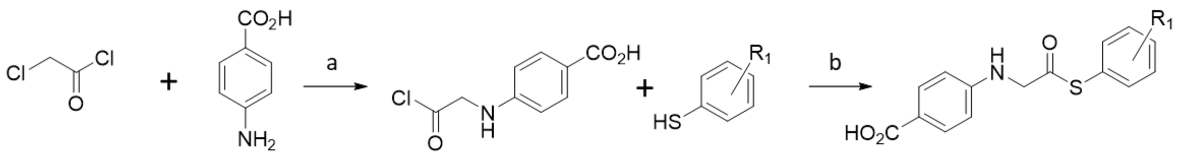

Scheme S1 compound labels: **1**, **2**, **3**, **4a** R_1 = 4-NO_2, **4b** R_1 = 2-CO_2H, **E1, E3**

Scheme S1. General synthetic procedure for the synthesis of compounds E1 and E3. (a): Na_2CO_3(aqu.), CH_3CN, 0 °C 30 min; (b): K_2CO_3/acetone, RT. 12 h.

4-((2-chloro-2-oxoethyl)amino)benzoic acid (3)

To a solution of p-aminobenzoic acid **2** (10,93 mmol, 1.0 eq.) and Na_2CO_3 (31.17 mmol, 2.85 eq.) in water (30 mL) at 0 °C under stirring, a solution of chloroacetylchloride (15,31 mmol, 1.4 eq.) in anhydrous CH_3CN (5mL) was added dropwise during 10 min. The suspension thus obtained was stirred at 0 °C for another 10 min, then warmed to RT for 10 min. Afterwards, the pH was adjusted (pH =1) with HCl 1 N, and the precipitate was thus obtained, collected and washed with water. The product was then purified by trituration with anhydrous diethyl ether. Yield 62 %. ^{1}H-NMR (DMSO-d_6, 400 MHz) (δ) (ppm): 10.65 (1H, broad s), 7.93 (2H, m), 7.71 (2H, m), 4.03 (2H, s); ^{13}C-NMR (DMSO-d_6, 101 MHz) (δ) (ppm): 167.2, 165.7, 143.0, 130.9, 126.2, 119.0, 30.6.

General procedure for the synthesis of 2-(Substituted aryl- or heteroarylthio)-N-(substituted phenyl)acetamide derivatives (E1, E3)

To a suspension of K_2CO_3 (3.0 eq.) in anhydrous acetone (20 mL) at RT under stirring in a N_2 atmosphere, the appropriate mercapto derivative (1.0 eq.) was added, followed by 4-((2-chloro-2-oxoethyl)amino)benzoic acid **3** (1 eq.).The suspension was stirred at RT for 12 hours, then the mixture was cooled to 0 °C, the pH adjusted with HCl 1N (pH 1.0) and the solid thus obtained was collected by filtration and washed with water. The crude product was purified by crystallization:

2-(4-Nitrophenylthio)-N-(4-carboxyphenyl)propionamide (E1): Yield 74%, (trituration with diethyl ether), ^{1}H NMR (400 MHz, DMSO) δ 4.14 (s, 2H), 7.55 – 7.64 (m, 2H), 7.67 (d, J = 8.4 Hz, 2H), 7.90 (d, J = 8.3 Hz, 2H), 8.11 – 8.20 (m, 2H), 10.71 (s, 1H). ^{13}C NMR (101 MHz, DMSO) δ 36.04, 118.42, 123.91, 126.43, 130.37, 142.30, 144.70, 146.79, 166.44. ESI-HRMS calc. for $C_{15}H_{13}N_2O_5S^+$ ($M+H^+$) 333,0545, found 333.0532. HPLC-UV/Vis t_r:13.961, purity: 97.9% λ = 220 nm.

2-Phenyl-2-(4-nitrophenylthio)-N-(4-carboxyphenyl)carboxyilic acid (E3): Yield 65%, (column chromatography CH_2Cl_2/CH_3OH 9.5:0.5), ^{1}H NMR (400 MHz, DMSO) δ 12.93 (s, 2H), 10.59 (s, 1H), 7.94 – 7.86 (m, 3H), 7.75 – 7.65 (m, 2H), 7.62 – 7.48 (m, 2H), 7.23 (m, 1H), 3.91 (s, 2H). ^{13}C NMR (101 MHz, DMSO) δ 167.44, 166.85, 142.87, 140.48, 132.50, 131.01, 130.46, 127.80, 125.66, 125.40,

124.31, 118.44, 48.60, 36.66. ESI-HRMS calc. for $C_{16}H_{14}NO_5S^+$ ($M+H^+$) 332,0593, found 332.0578. HPLC-UV/Vis t_r:10.826, purity: 97.4% λ = 220 nm.

Optimized procedure for the synthesis of E7

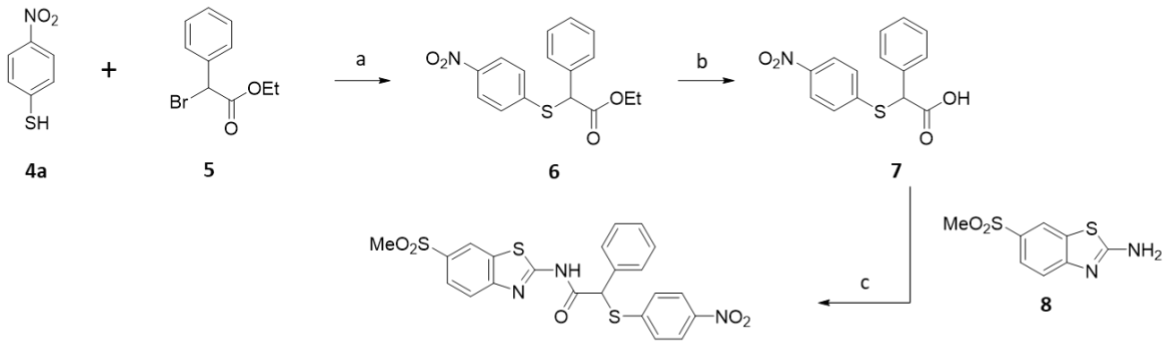

Scheme S2. General synthetic procedure for the synthesis of compounds E7. (a): K_2CO_3/acetone, RT. 1 h; (b): NaOH 1M, RT, 3h, THF:CH_3OH; (c): HATU, DIPEA, RT, 12h, DMF.

ethyl-2-[(4-nitrophenylthio)]acetate (6)

4-nitrothiophenol **4a** (12.9 mmol, 1.0 eq.) and K_2CO_3 (15.47 mmol. 1.2 eq.) were suspended in anhydrous acetone (20 mL) under N_2 atmosphere at RT. To the suspension, ethyl alpha-bromophenylacetate **5** (15.47 mmol, 1.2 eq.) was added dropwise, and the suspension was stirred for 1 hour. At the end of the reaction, water was added (40 mL) and the mixture was extracted with EtOAc (3x10 mL); the organic phase was collected, filtered, and dried (Na_2SO_4) and the solvent removed under reduced pressure. Yield 95%, oil, ^{1}H-NMR ($CDCl_3$, 400 MHz) (δ) (ppm): 8.11 (m, 2H), 7.54 (dd, *J* = 8.0 Hz, *J* = 2.0 Hz, 2H), 7.40 (m, 5H), 4.20 (m, 3H), 1.33 (t, J = 7.2 Hz, 3H); ^{13}C-NMR ($CDCl_3$, 100 MHz) (δ) (ppm): 169.5, 146.1, 144.5, 134.3, 129.0, 128.9, 128.9, 128.4, 123.9, 62.3, 54.6, 14.0.

2-Phenyl(4-nitrophenylthio)]acetic acid (7)

To a solution of ester **6** (6.87 mmol, 1.0 eq.) in a mixture of THF:CH_3OH (15:5 mL) aqueous NaOH 1M (10.31 mmol, 1.5 eq.) was added and the solution thus obtained was stirred at RT under for 3 h. At the end of the reaction (TLC), the solvent was removed under reduced pressure. The residue thus

obtained was dissolved in H_2O and washed with DCM (3x), and the water phase was collected and pH adjusted (pH =1) with HCl 1 N. The solid obtained was filtered; the product was used without further **purification.** Yield 96%. ^{1}H-NMR (DMSO-d$_6$, 400 MHz) (δ) (ppm): 8.27 (m, 2H), 7.74–7.66 (m, 4H), 7.55–7.45 (m, 3 H), 5.79 (s, 1 H); ^{13}C-NMR (DMSO-d$_6$, 100 MHz) (δ) (ppm): 171.1, 145.7, 145.6, 135.9, 129.3, 128.8, 128.2, 124.3, 53.2.

phenyl-2-(4-nitrophenylthio)-N-(6-methanesulfonylbenzothiazol-2-yl)acetamide (E7)

2-Phenyl(4-nitrophenylthio)]acetic acid **7** (3.33 mmol, 1.0 eq.) was dissolved in anhydrous DMF (10 mL) under stirring in a N_2 atmosphere and the solution cooled to 0 °C. To this solution were added HATU (3.66 mmol, 1.1 eq.), 2-amino-6-methanesulphonylbenzothiazole **8** (3.33 mmol, 1.1 eq.) and DIPEA (9.99 mmol, 3.0 eq.) in sequence and the mixture was warmed to RT and stirred for 12h. After 12 h at RT the solvent was removed under vacuum. The residues were dissolved in EtOAc and washed in sequence with a solution of HCl 1M (3x), $NaHCO_3$ (3x) and brine (3x). The organic layers were collected, filtered and dried (Na_2SO_4). The solvent was removed under reduced pressure. Firstly, the solid thus obtained was purified by trituration with a mixture of DCM and CH_3OH with the aid of ultrasound followed by FC (CH_2Cl_2:CH_3OH 9.5:0.5). Yellow solid, yield 40%, m.p. 95 °C (dec.). ^{1}H NMR (600 MHz, DMSO) δ 13.35 (s, 1H), 8.72 – 8.61 (m, 1H), 8.20 – 8.14 (m, 2H), 7.98 – 7.92 (m, 2H), 7.66 – 7.58 (m, 2H), 7.59 – 7.53 (m, 2H), 7.43 (t, *J* = 7.6 Hz, 2H), 7.40 – 7.32 (m, 1H), 5.83 (s, 1H), 3.24 (s, 3H).; ^{13}C-NMR (DMSO-d$_6$, 50 MHz) (δ) (ppm): 174.01, 168.10, 152.09, 146.03, 136.30, 134.99, 129.52, 129.35, 128.93, 128.67, 125.44, 124.67, 122.72, 121.62, 53.98, 44.46. ESI-HRMS calc. for $C_{22}H_{18}N_3O_5S_3$ (M+H$^+$) 500.0390, found 500.0404. HPLC-UV/Vis t_R:14.60 min, purity 98.7%.

Table S1. SQ values ± SD of the synergism quotient analysis to evaluate increased efficacy obtained by dividing the result of the inhibition of the combination of compounds E1 and E7 with SAINT-Protein by the sum of the inhibition of the single component. A quotient> 1 indicates synergism, while a quotient <1 or = 1 indicates an antagonistic or additive effect, respectively.

Molecules	A2780 cells	A2780/CP cells
**E1 12.5 µM**	1.14±0.1	1.22±0.1
**E1 25 µM**	1.22±0.1	1.90±0.6
**E1 50 µM**	1.02±0.1	1.71±0.4
**E7 2.5 µM**	1.36±0.2	1.90±0.1

**E7 5 µM**	1.10±0.05	1.54±0.45
**E7 12 µM**	1.07±0.01	1.34±0.1
**E7 25 µM**	0.90±0.1	1.15±0.03

Table S2. E1, E3, E5-E7 chemico-physical properties calculation.

E1, E3, E5-E7 chemico-physical properties have been calculated using MarvinSketch[2] calculator plugins[3]. LogP calculations have been performed using the ChemAxon method and considering electrolyte concentration of Cl^-, K^+ and Na^+ of 0,1 mol/dm^3. Log D has been calculated using LogP with the same specific and considering lower pH value = 0 and upper pH value = 14 with a pH step size of 0.5. Four pH values have been considered: 1.5, 5, 6.5 and 7.4.

<table>
<tr><th>Entry</th><th>Structure</th><th>MW</th><th>LogP</th><th>LogD</th><th>Water solubility
pH = 7.4</th></tr>
<tr><td>E1</td><td>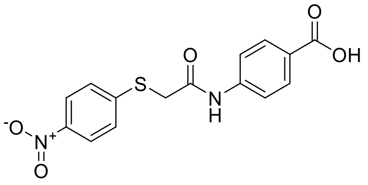</td><td>332.33</td><td>2.93</td><td><table><tr><td>pH</td><td>log D</td></tr><tr><td>1.50</td><td>2.93</td></tr><tr><td>5.00</td><td>2.03</td></tr><tr><td>6.50</td><td>0.61</td></tr><tr><td>7.40</td><td>-0.18</td></tr></table></td><td>5.90 mg/mL</td></tr>
</table>

<table>
<tr><td>E3</td><td>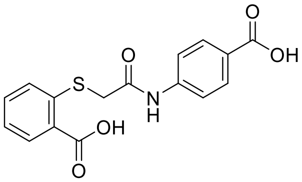</td><td>331.34</td><td>2,49</td><td><table><tr><td>pH</td><td>log D</td></tr><tr><td>1.50</td><td>2.49</td></tr><tr><td>5.00</td><td>1.15</td></tr><tr><td>6.50</td><td>-1.59</td></tr><tr><td>7.40</td><td>-3.23</td></tr></table></td><td>331.34 mg/mL</td></tr>
<tr><td>E5</td><td>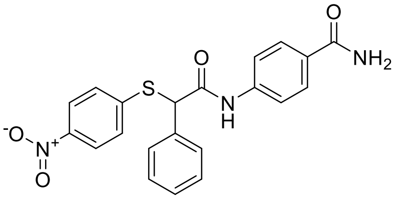</td><td>407.45</td><td>4.04</td><td><table><tr><td>pH</td><td>log D</td></tr><tr><td>1.50</td><td>4.02</td></tr><tr><td>5.00</td><td>4.04</td></tr><tr><td>6.50</td><td>4.04</td></tr><tr><td>7.40</td><td>4.04</td></tr></table></td><td><0.00010 mg/mL</td></tr>
<tr><td>E6</td><td>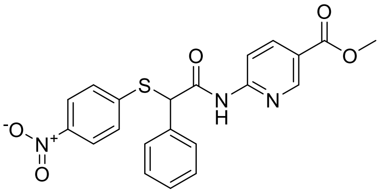</td><td>423.449</td><td>4.92</td><td><table><tr><td>pH</td><td>log D</td></tr><tr><td>1.50</td><td>4.20</td></tr></table></td><td><0.00010 mg/mL</td></tr>
</table>

				5.00	4.92	
				6.50	4.92	
				7.40	4.92	
**E7**	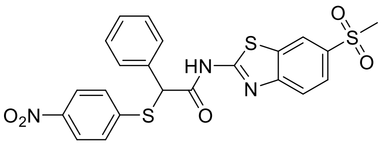	499.592	4.89	pH	log D	<0.0010 mg/mL
				1.50	4.89	
				5.00	4.89	
				6.50	4.87	
				7.40	4.73	

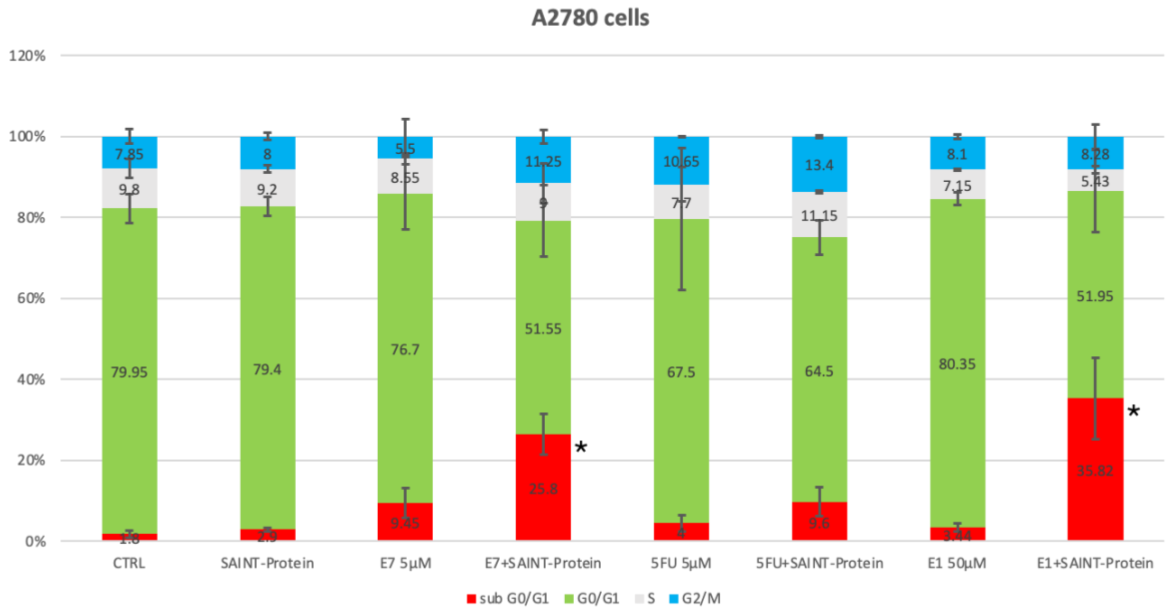

Figure S1. Cell cycle phase distribution analysis of A2780 Cells. Effect of the combination of the Ddis drugs with SAINT-Protein for 72 hr on the distribution of cell cycle phases in the A2780 OC cell line by flow cytometric analysis of DNA content with PI staining. A2780 cells treated with E1, E7 and 5FU with or without SAINT-Protein. Inserted numbers indicate the % of cells in the different phases of the cell cycle. Experiments have been performed in replicate. Error bars, SD. * $p \leq 0.05$, ** $p < 0.01$, in comparison to non-transfected drug samples.

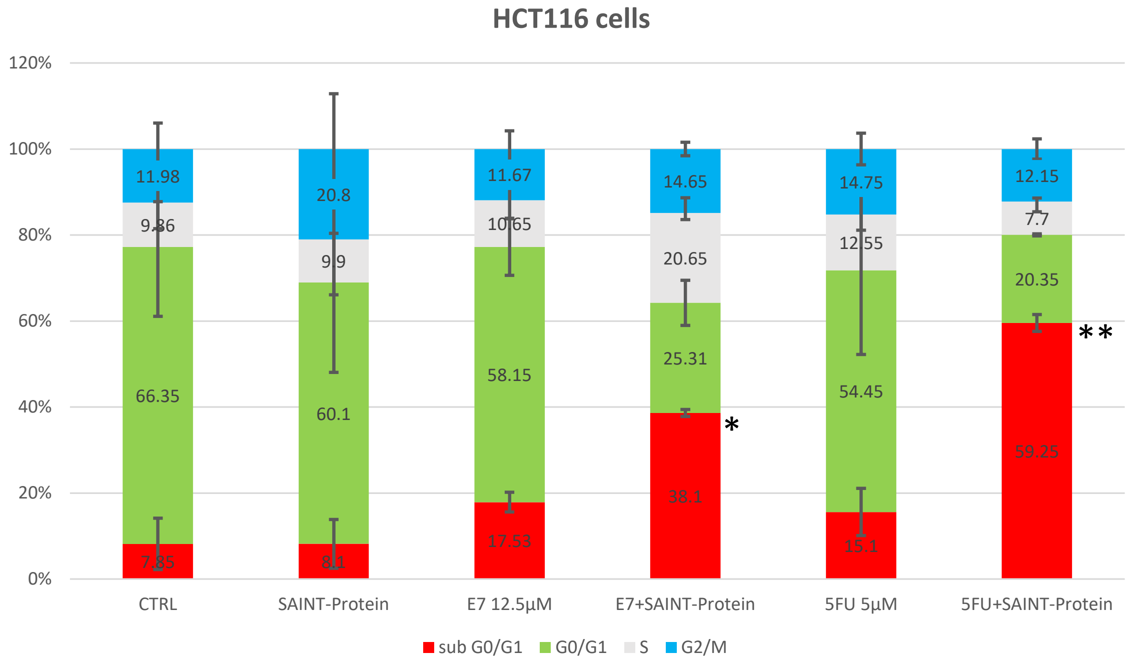

Figure S2. Cell cycle phase distribution analysis of HCT116 Cells. Effect of the combination of the Ddis with SAINT-Protein for 72 hr on the distribution of cell cycle phases in the HCT116 cell line by flow cytometric analysis of DNA content with PI staining. HCT116 cells were treated with E7 and 5FU with or without SAINT-Protein. Inserted numbers indicate the % of cells in the different phases of the cell cycle. Experiments have been performed in replicate. Error bars, SD. * $p \leq 0.05$, ** $p < 0.01$, in comparison to non-transfected drug samples.

Scheme S3. hTS intact Mass Spectrometry protein analysis

Intact protein was analysed with an UltiMate 3000 UHPLC couple to an Orbitrap Exactive 480 (Thermo Fisher) High Resolution Mass Spectrometry (HRMS) by an ESI source. We used a MS method for spectra acquisition (450-5000 m/z), SID (in source CID) 40% NCE, 800ms Max IT, 3.6x10 3 AGCwith a BioShell IgG C4, 1000Å pore size, 2.1 x 100mm, 2.7µm particle size (Merck Millipore) column, thermostatted at 70°C (Merck Millipore). A 10% SID was applied. Each sample (10µM hTS) was diluted 10-fold with MilliQ water to obtain a concentration of about 1uM, and 6uL were injected into the system. No formic acid was added to prevent protein precipitation before entering the column. 0.1% aqueous Formic acid (FA), (A), and 0.1% FA in acetonitrile (B) were used as mobile phases. Gradient was set as in table 1, with a constant flow rate of 0.5mL/min. Samples were analyzed in duplicate with FreeStyle software (Thermo Fisher). Extracted ion chromatograms were submitted to the deconvolution algorithm provided by the vendor, which revealed a monoisotopic mass of 37,012.87 Da, consistent with the 37’012.81 Da of the theoretical model.

Table S3. UHPLC Gradient for hTS sample analysis.

**Time (min)**	**%B**	**Flow rate (mL/min)**
0	10	0.5
45	92	0.5
50	92	0.5
50.1	10	0.5
60	10	0.5

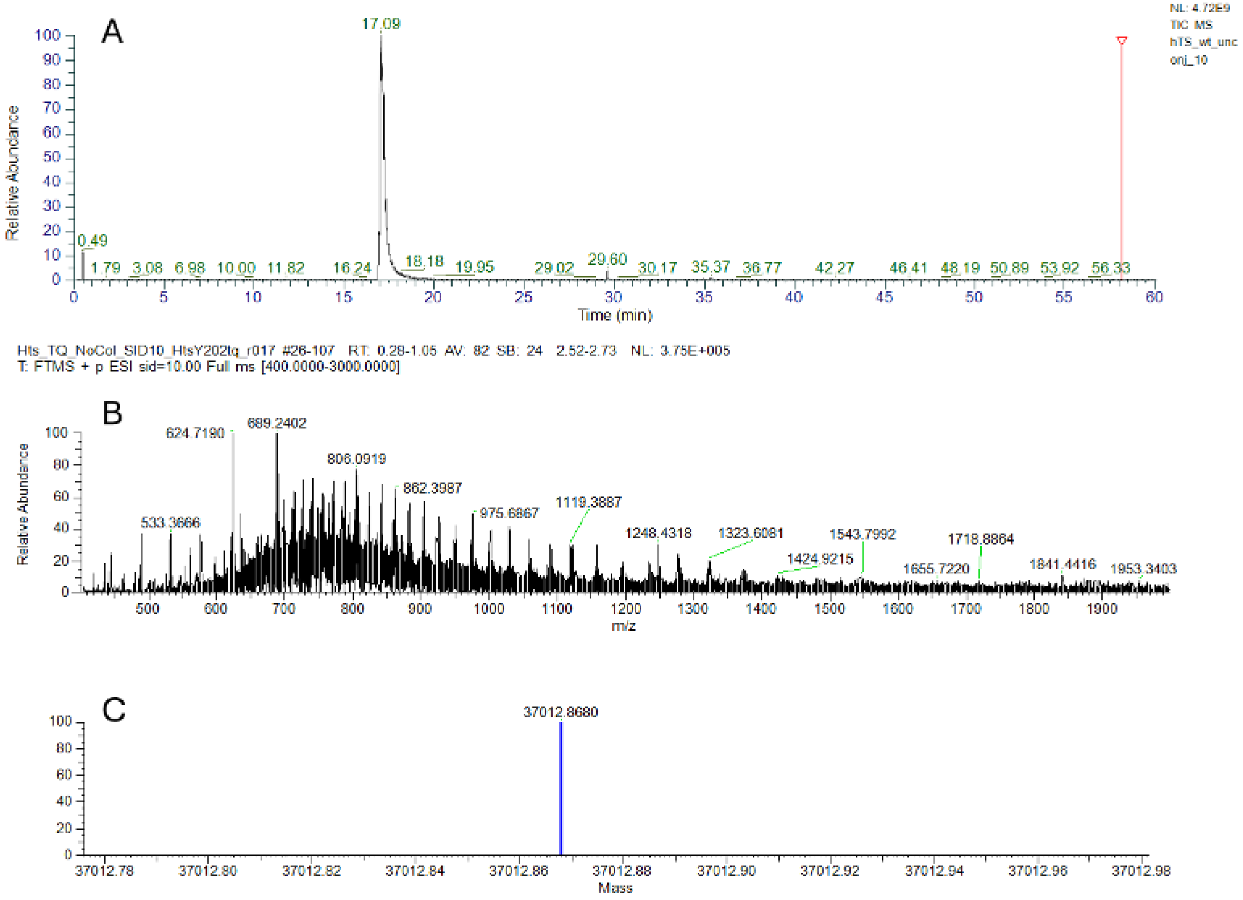

Figure S3. (A) hTS wt LC-MS Full-MS TIC (total ion current) chromatogram. (B). FullMS Spectrum of peak rt 17.09 from A. (C). Bayesian deconvolution with FreeStyle (ThermoFisher) confirmed *h*TS MW (monoisotopic expected mass: 37’012.81 Da; monoisotopic experimental mass: 37’012.87 Da).

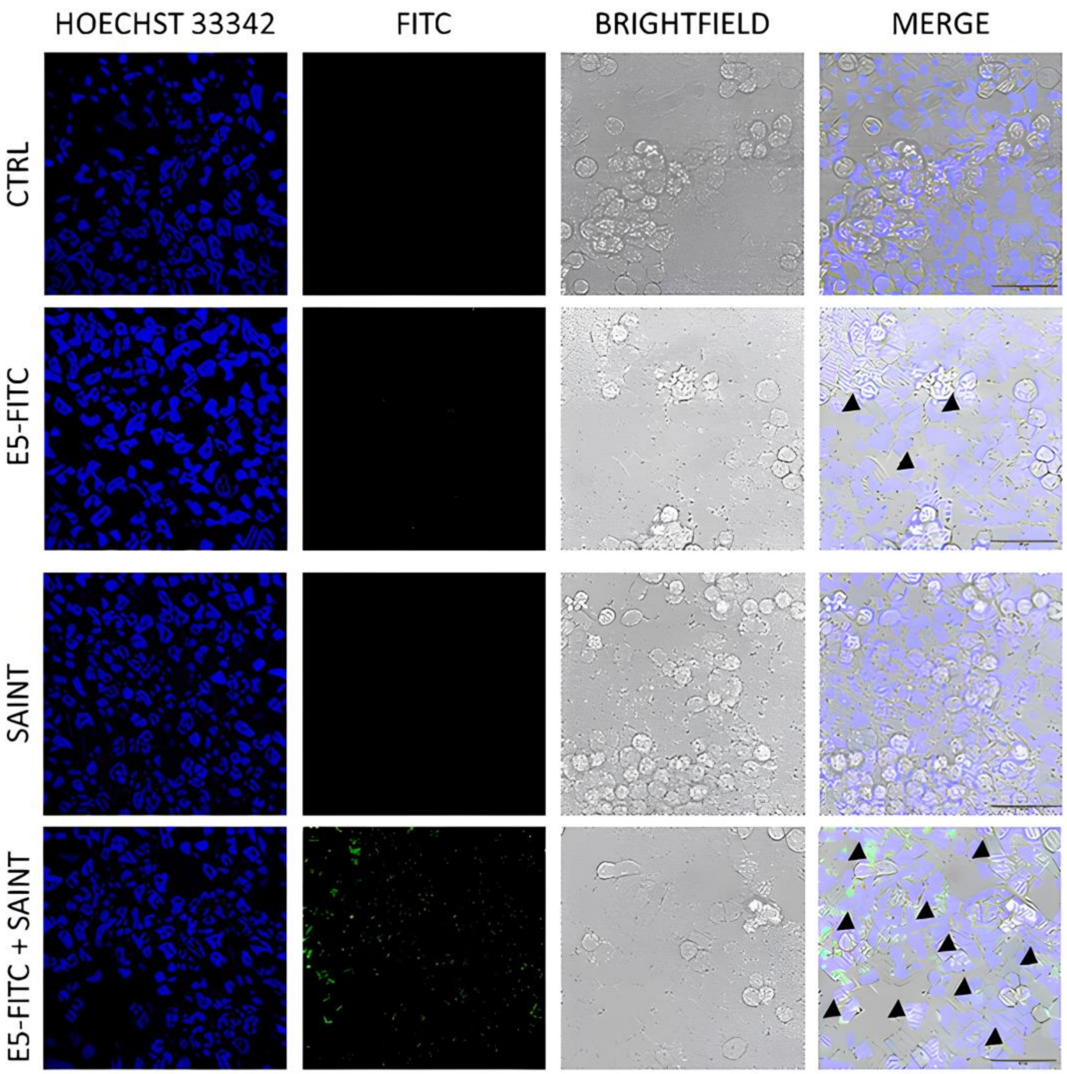
HOECHST 33342
FITC
BRIGHTFIELD
MERGE
CTRL
E5-FITC
SAINT
E5-FITC + SAINT

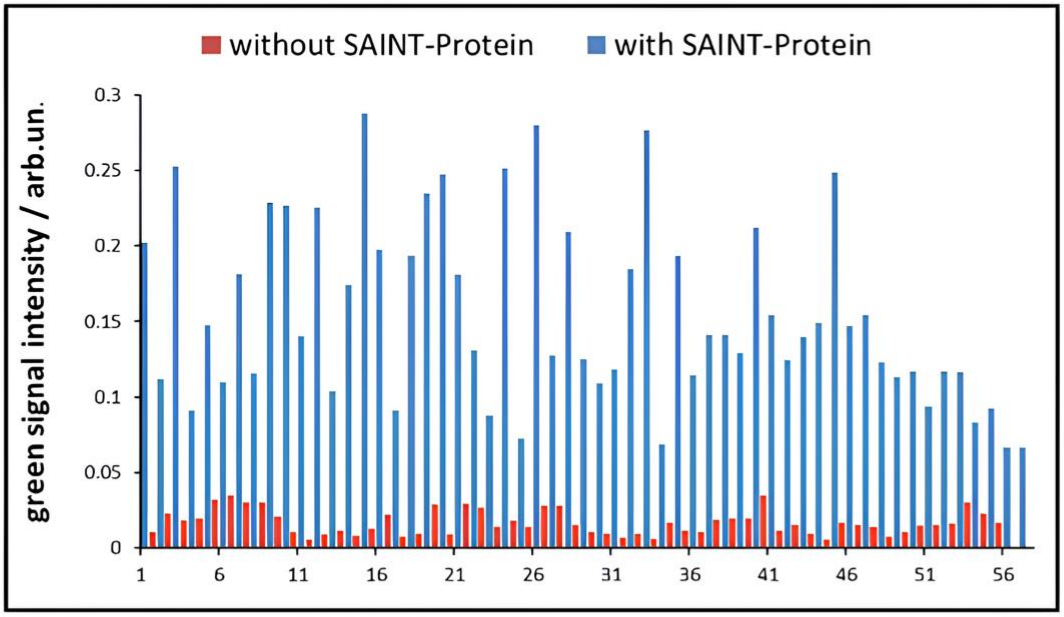
without SAINT-Protein
with SAINT-Protein
green signal intensity / arb.un.
0.3
0.25
0.2
0.15
0.1
0.05
0
1
6
11
16
21
26
31
36
41
46
51
56

Figure S4. Representative pictures obtained by confocal fluorescence microscopy imaging of living cells A2780. The pictures and the quantification graf show the increase in cellular uptake of our compound E5-FITC conjugated caused by the SAINT-Protein transfection agent. Cellstain-Hoechst 33342 solution-nucleus dye in blue, FITC fluorescent dye (E5-FITC) in green. Results of the experiments are expressed as mean ± standard error of the mean (S.D). All mean differences were considered statistically significant if *p< 0.05; **p<0.01; ***p< 0.001. scale bar: 50 μm.

References

[1]. Luca Costantino, Stefania Ferrari, Matteo Santucci, Outi MH Salo-Ahen, Emanuele Carosati, Silvia Franchini, Angela Lauriola, Cecilia Pozzi, Matteo Trande, Gaia Gozzi, Puneet Saxena, Giuseppe Cannazza, Lorena Losi, Daniela Cardinale, Alberto Venturelli, Antonio Quotadamo, Pasquale Linciano, Lorenzo Tagliazucchi, Maria Gaetana Moschella, Remo Guerrini, Salvatore Pacifico, Rosaria Luciani, Filippo Genovese, Stefan Henrich, Silvia Alboni, Nuno Santarem, Anabela da Silva Cordeiro, Elisa Giovannetti, Godefridus J Peters, Paolo Pinton, Alessandro Rimessi, Gabriele Cruciani, Robert M Stroud, Rebecca C Wade, Stefano Mangani, Gaetano Marverti, Domenico D'Arca, Glauco Ponterini, Maria Paola Costi **(2022)** Destabilizers of the thymidylate synthase homodimer accelerate its proteasomal degradation and inhibit cancer growth *eLife* 11:e7386. https://doi.org/10.7554/eLife.73862

[2] "Marvin was used for drawing, displaying and characterizing chemical structures, substructures and reactions, Marvin 24.1.1**, 2024,** ChemAxon (http://www.chemaxon.com)".

[3] "Calculator Plugins were used for structure property prediction and calculation, Marvin 24.1.1, **2024,** ChemAxon (http://www.chemaxon.com)" https://docs.chemaxon.com/display/docs/calculators_logd-plugin.md.
